# SPDL1 Is an Independent Predictor of Patient Outcome in Colorectal Cancer

**DOI:** 10.3390/ijms23031819

**Published:** 2022-02-05

**Authors:** Anna Klimaszewska-Wiśniewska, Karolina Buchholz, Justyna Durślewicz, Emilly Schlee Villodre, Maciej Gagat, Dariusz Grzanka

**Affiliations:** 1 Department of Clinical Pathomorphology, Faculty of Medicine, Collegium Medicum in Bydgoszcz, Nicolaus Copernicus University in Torun, 85-094 Bydgoszcz, Poland; karolina.buchholz@cm.umk.pl (K.B.); justyna.durslewicz@cm.umk.pl (J.D.); d_grzanka@cm.umk.pl (D.G.); 2 Department of Histology and Embryology, Faculty of Medicine, Collegium Medicum in Bydgoszcz, Nicolaus Copernicus University in Torun, 85-092 Bydgoszcz, Poland; mgagat@cm.umk.pl; 3 Department of Breast Medical Oncology and MD Anderson Morgan Welch Inflammatory Breast Cancer Clinic and Research Program, The University of Texas MD Anderson Cancer Center, Houston, TX 77030, USA; ESSchlee@mdanderson.org

**Keywords:** colorectal cancer, SPDL1, prognostic factor, genomic instability

## Abstract

Spindle Apparatus Coiled-Coil Protein 1 (SPDL1) is a relatively recently identified coiled-coil domain containing protein and an important determinant of DNA fidelity by ensuring faithful mitosis. Hence, SPDL1 is suspected to underlie genomic (in-)stability in human cancers, yet its exact roles in these diseases remain largely underexplored. Given that genomic instability (GIN) is a crucial feature in colorectal cancer (CRC), we primarily asked whether the expression of this protein may account for differences in clinicopathological features and survival rates of CRC patients. Protein expression was evaluated by immunohistochemistry in the institutional tissue microarray (TMA), and gene expression by the analysis of publicly available datasets. To place the prognostic relevance in a predicted biological context, gene co-expression set around SPDL1 identified by public data mining was annotated and assessed for enrichment in gene ontology (GO) categories, BRITE hierarchies, and Reactome pathways. The comparison with adjacent normal tissue revealed a high expression of SPDL1 protein in a subset of tumor cases (48.84%), and these had better prognosis than the SPDL1-low expression counterpart even after adjustment for multiple confounders. SPDL1-high expression within tumors was associated with a median 56-month survival advantage, but not with any clinicopathological characteristics of our cohort. In the TCGA cohort, *SPDL1* was overexpressed in tumor tissue and positively associated with improved survival, chromosome instability phenotype, and various GIN markers. In addition to the genes critically involved in the cell cycle and mitosis, a gene set co-expressed with *SPDL1* contained checkpoint members of both chromosome segregation and DNA replication, as well as those associated with defective DNA repair, and retrograde vesicle-mediated transport. In conclusion, SPDL1 is an independent predictor of CRC patient survival in a possible connection with chromosomal instability.

## 1. Introduction

According to GLOBOCAN 2020 data, carcinoma of the colon or rectum (colorectal cancer [CRC]) is the third most commonly diagnosed cancer, and the second leading cause of cancer death worldwide, despite progress made in diagnosis, prognosis and management through surgery, radiotherapy, chemotherapy, and immunotherapy [1,2]. Even with these advances, there is a striking scarcity of biomarkers that can guide the early detection, prognostic stratification, targeted treatment, and surveillance of CRC patients highlighting the need for further investigation [2].

Genomic instability (GIN) plays an active role in the development of CRC by creating a permissive environment for tumor’s adaptation to external challenges and selective pressure and hence, for continued survival fitness. Molecular evidence from GIN and abnormal gene/protein expression has recently emerged as a rich resource to identify biomarkers for CRC [3], and other tumors as well [4]. There are two major categories of GIN in CRC: chromosome instability (CIN) and microsatellite instability (MSI), whereby–etiologically, CIN (80–85%) is more prevalent than MSI (15–20%). Molecular causes for the MSI phenotype are defects in DNA replication and repair systems, while CIN is caused mainly by failures in the mitotic process and/or in the mitotic apparatus [3]. Herein, we focused on a candidate protein–Spindle Apparatus Coiled-Coil Protein 1 (SPDL1), as an important determinant of DNA fidelity through ensuring faithful mitosis.

SPDL1 encodes Spindly (also known as CCDC99), a coiled-coil domain containing protein that was identified in 2007 in Drosophila and human cells as a new kinetochore regulator of dynein, being essential for silencing the spindle assembly checkpoint [5]. After the initial study, its critical role in mitotic spindle formation and chromosome segregation has been supported by further evidence, and, in addition, SPLD1′s interphase function was proposed [6]. With regard to the latter, this protein was shown to be required for rapid migration of human cells, likely due to its ability to regulate dynein/dynactin activity, similar as in mitosis [6]. However, the role of SPDL1 in tumorigenesis is poorly understood, and very few studies, including our recent one [7] had addressed this issue [8,9,10,11]. Specifically, as far as we know, no studies have been conducted in CRC to evaluate its clinical value at the protein level. 

Given that GIN has an important contribution to the pathology of CRC, the present study was designed to determine the clinical value of SPDL1 in CRC. We evaluated the expression, clinicopathological associations, and survival implications of SPDL1 in the institutional tissue microarrays (TMA) and publicly available datasets. Furthermore, the biological significance of SPDL1 was captured based on the functional enrichment analyses through online bioinformatics platforms and web tools. 

## 2. Results

### 2.1. Immunoexpression of SPDL1 in Tumor and Normal Adjacent Tissue and Its Clinicopathological Associations

SPDL1 expression was predominantly cytoplasmic in CRC, with several tissues showing combined cytoplasmic-membranous (9.30%) or cytoplasmic-nuclear (6.98%) expression. Cytoplasmic SPDL1 was characterized by ubiquitous, diffuse staining on tumor cells but not in a uniform intensity. Only a few cells within the tumor gland displayed nuclear staining pattern. In non-cancerous tissues, staining was fully restricted to the cytoplasm. Representative images showing immunostaining for SPDL1 in tumor and adjacent tissues are presented in Figure S1. 

**Figure 1:**
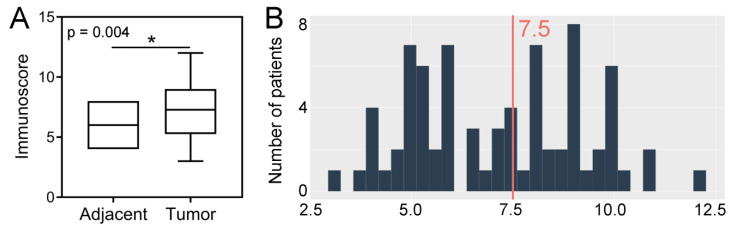
(**A**) Immunohistochemical expression of SPDL1 in colorectal cancer compared to non-cancerous adjacent tissues. The error bars present the range from minimum to maximum values of data. Asterisk (*) indicates statistically significant difference (*p* < 0.05). (**B**) The optimal cut-off value established for SPDL1 based on IRS scores and Evaluate Cutpoints software.

The expression levels of SPDL1 were significantly high in CRC tissues compared to the adjacent non-cancerous tissues (*p* = 0.004; Figure 1A). Based on the established cut-off point (Figure 1B), SPDL1-high was found in 42 (48.84%) CRC tissues, as well as in 9 (33.33%) non-cancerous tissues. However, the expression status of SPDL1 was not associated with any examined clinicopathological data listed in Table 1 (*p* > 0.05), including age, gender, grading, primary tumor (pT) classification, regional lymph node (pN) status, distant metastasis (pM) status, AJCC TNM stage, vascular invasion, perineural invasion, resection margins, and primary tumor location. 

### 2.2. Prognostic Value of SPDL1 Immunoexpression in Predicting the Overall Survival of CRC Patients

**Figure 2:**
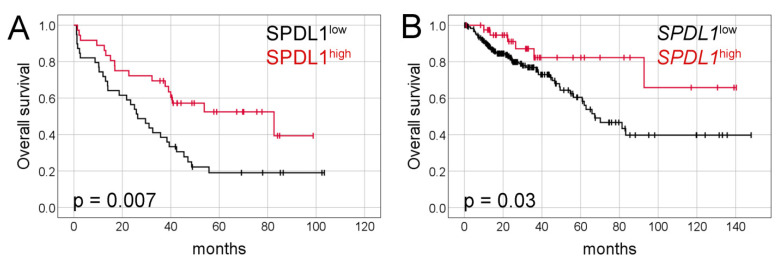
Kaplan-Meier survival curves and log-rank test for overall survival of CRC patients based on (**A**) SPDL1 protein expression and (**B**) SPDL1 mRNA expression.

Kaplan-Meier analysis demonstrated that the median overall survival (OS) was significantly longer in patients with SPDL1-high tumors than in those with SPDL1-low tumors (82 months, 95% confidence interval [CI] 21.9–143.4 vs. 26 months, 95% CI 17.0–35.6, respectively, *p* = 0.007, Figure 2A).

In the univariate Cox model, SPDL1-high was significantly related to a favorable survival rate (hazard ratio [HR] = 0.45, 95% CI 0.25–0.82, *p* = 0.009; Table 2). However, CRC patients with poorly differentiated (HR = 2.58, 95% CI 1.00–6.69, a borderline *p* value = 0.05) or late-stage tumors (HR = 2.93, 95% CI 1.37–6.27, *p* = 0.006), or those experiencing distant metastasis (HR = 2.81, 95% CI 1.55–5.08, *p* = 0.001), or positive resection margin (HR = 1.86, 95% CI 0.98–3.53, a borderline *p* value = 0.057) had markedly poorer prognosis (Table 2). 

In the multivariate Cox model, SPDL1 (HR = 0.42, 95% CI 0.23–0.78, *p* = 0.006), tumor grade (HR = 3.87, 95% CI 1.36–11.01, *p* = 0.01), pM stage (HR = 3.32, 95% CI 1.73–6.37, *p* = 0.0003), resection margin (HR = 2.37, 95% CI 1.13–4.98, *p* = 0.02) were found to be independent prognostic factors predicting OS of CRC patients (Table 2). After adjustment for multiple confounders, the strength of association for tumor location increased and was of borderline significance (rectum vs. right colon HR = 2.10, 95% CI 1.00–4.44, *p* = 0.05; Table 2). Separate multivariate Cox model was built under the same clinical variables setting as shown in Table 2 except including AJCC TNM stages instead of the pT, pN, pM categories (Table S1). It was demonstrated that SPDL1 remained significantly associated with OS after adjusting for age, gender, grading, resection margin, AJCC TNM stage, and tumor location. 

To verify the stability of our findings, we additionally explored the prognostic value of SPDL1 with the IRS and IS as continuous variables in the univariate and multivariate Cox models, and found it remained significant for OS in both analyses (Table S2). We then restricted our survival analysis to individuals with complete information (no missing data), and there was still a clear association between SPDL1 and OS (Figure S2A, Table S3).

For additional survival analysis, SPDL1 expression was split into three groups to make further distinction between low and intermediate IRS scores. This analysis showed that both low expression group (39 months, 95% CI 36.6–42.2) as well as high expression group (82 months 95% CI 21.9–143.4) had better median OS than moderate expression group (21 months 95% CI 7.0–36.5; *p* = 0.01; Figure S2B). 

We also examined the subcellular distribution of SPDL1 in relation to OS of CRC patients. However, we did not find any difference in OS between patients whose tumors exhibited SPDL1 staining confined exclusively to the cytoplasm compared to those with mixed pattern (nuclear and cytoplasmic or membranous and cytoplasmic) of SPDL1 expression (*p* = 0.57; Figure S2C).

We next analyzed survival according to SPDL1 expression in different AJCC TNM stage subgroups. Given the relatively small number of cases, cancer stages II and III were combined into one clinical subcohort. In this subgroup, there was a clear trend toward the association of SPDL1-high with better OS (*p* = 0.08; HR = 0.44, 95% CI 0.17–1.13; Figure S2D). Median duration of OS was 82 months (95% CI 32.5–132.8) in the high expression group, whilst it was 42 months (95% CI 30.0–54.8) for the low expression group. A similar trend was observed in stage IV CRCs (37 months, 95% CI 7.7–67.7 vs. 21 months, 95% CI 7.6–36.0; *p* = 0.09; HR = 0.52, 95% CI 0.24–1.12; Figure S2E). We also analyzed the OS stratified according to the expression of SPDL1 and the metastatic status of patients at diagnosis (Figure S2F), and found that the status of SPDL1-high and SPDL1-low could separate the Kaplan-Meier curves for patients with or without distant metastasis (*p* value pooled over strata = 0.01; *p* values for each stratum = 0.09 and 0.057, respectively). The optimal cut-off value for SPDL1 expression in each analyzed subgroup was the same as in the entire cohort (7.5).

### 2.3. TCGA CRC Cohort Analysis–Tissue Expression, Clinicopathological and Survival Associations, Correlations with GIN

**Figure 3:**
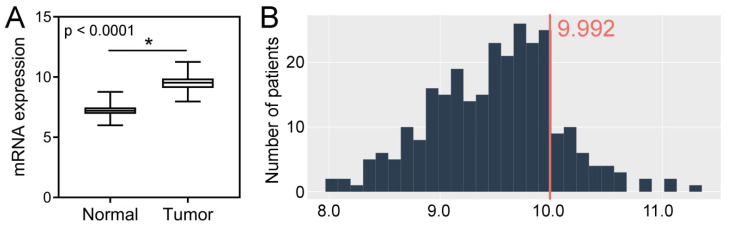
(**A**) The expression level of *SPDL1* mRNA in colorectal cancer compared to normal tissues. Analysis was performed based on gene expression data sourced from The Cancer Genome Atlas (TCGA) and Genotype-Tissue Expression (GTEx) databases. The error bars present the range from minimum to maximum values of data. Asterisks (*) indicate statistically significant differences (*p* < 0.05). (**B**) The optimal cut-off value for *SPDL1* mRNA expression level was established using Evaluate Cutpoints software.

Analysis of the TCGA dataset revealed that the expression levels of *SPDL1* were significantly higher in CRC tissues compared to the control group (*p* < 0.0001; Figure 3A). Based on the established cut-off value (Figure 3B), overexpression of SPDL1 was observed in 41 (14.9%) CRC cases. Moreover, the expression level of *SPDL1* was not associated with any examined clinicopathological features (*p* > 0.05; Table 3).

*SPDL1* expression levels in chromosomal instability (CIN subtype) tumors were significantly higher compared to genomically stable (GS subtype) tumors (*p* < 0.0001; Figure S3A), as well as in microsatellite instability (MSI) tumors compared to GS tumors (*p* = 0.002; Figure S3B). Accordingly, *SPDL1* was positively correlated with CIN (r = 0.30; *p* < 0.0001) and MSI (r = 0.33; *p* = 0.002). *SPDL1* was also associated with aneuploidy score (r = 0.13; *p* = 0.03), but not with mutation count (r = 0.10; *p* = 0.12), MSI MANTIS score (r = 0.01; *p* = 0.90) and MSI sensor score (r = 0.04; *p* = 0.54). Furthermore, it was correlated with *MKI67* (r = 0.56; *p* < 0.0001), *MSH6* (r = 0.58; *p* < 0.0001), *MSH2* (r = 0.58; *p* < 0.0001), *MLH1* (r = 0.35; *p* < 0.0001), *PMS2* (r = 0.35; *p* < 0.0001), as well as with the expression of almost all 25 genes associated with functional aneuploidy (CIN25 signature) [12], including *FOXM1* (r = 0.53; *p* < 0.0001). Of the CIN25 genes, *SPDL1* was not significantly correlated with *TP53* gene (Figure S4).

Kaplan-Meier survival analysis demonstrated that CRC patients with *SPDL1*-high transcript expression survived markedly longer than those with *SPDL1*-low (not reached vs. 67 months, 95% CI 48.3–86.3, *p* = 0.03; Figure 2B). The favorable prognostic effects of *SPDL1* were also observed in the univariate and multivariate Cox analyses (crude HR = 0.41, 95% CI 0.18–0.96; *p* = 0.04; adjusted HR= 0.37, 95% CI 0.16–0.85; *p* = 0.02; Table 4). Separate multivariate Cox model was built under the same clinical variables setting as shown in Table 4 except including the TNM stages instead of the pT, pN, pM categories (Table S4). It was demonstrated that *SPDL1* remained significantly associated with OS after adjusting for age, gender, and tumor stage. 

When analyzed only in AJCC stage II CRCs, no significant difference was observed regarding OS between *SPDL1* expression groups (not reached vs. 81 months, 95% CI 65.0–97.7; *p* = 0.30; HR = 0.52, 95% CI 0.15–1.83; Figure S5A). However, a cutoff value of 9.02 more accurately differentiated stage II patients in OS than that established for the entire cohort (9.992). Following cutoff value optimization, *SPDL1*-high stage II cases were associated with a significantly better OS (not reached vs. 62 months, 95% CI 58.1–67.5; *p* = 0.04; HR = 0.40, 95% CI 0.16–1.00; Figure S5B). A significant difference in OS was also observed for the subcohort containing stages III and IV tumors (not reached vs. 45 months, 95% CI 24.8–66.0; *p* = 0.02; HR = 0.28, 95% CI 0.09–0.89; Figure S5C). Cancer stages III and IV were analyzed together due to a limited size of the patients in stage IV; this was also why we failed to analyze OS stratified according to the expression of *SPDL1* and the metastatic status of TCGA patients at diagnosis. 

### 2.4. Coexpressed Genes and Functional Enrichment Analyses

**Figure 4:**
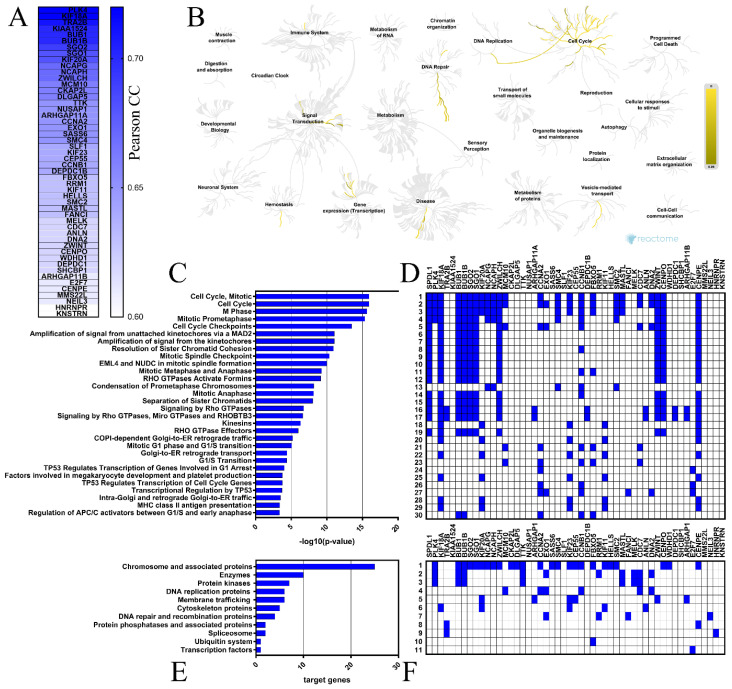
Functional enrichment analyses for the top 50 genes positively correlated with *SPDL1* in CRC. (**A**) These genes are depicted with the corresponding Pearson correlation coefficient (Pearson CC) values. (**B**) The Reactome Pathway hierarchy panel. (**C**) Bar chart of the top 30 Reactome pathways based on the enrichment scores [−log10 (*p*-value)]. (**D**) Heatmap of the top 30 Reactome pathways based on the target genes. (**E**) Bar chart for all terms in BRITE functional hierarchies. The horizontal axis represents the number of genes enriched in KEGG Brite terms represented on the vertical axis. (**F**) Heatmap for all terms in BRITE functional hierarchies based on the target genes.

The top 50 genes that were positively correlated with *SPDL1* in colon cancer tissues were determined based on the TCGA data using the UALCAN web-based tool (Figure 4A), then verified for the expression profile in both colon and rectal cancers through GEPIA database, and confirmed to be significantly upregulated in tumors compared to normal tissues (data not shown). Polo Like Kinase 4 (*PLK4*) and Kinesin Family Member 18A (*KIF18A*) had the highest positive correlation with *SPDL1* (Pearson correlation coefficient r = 0.72; Table S5). Correlation analysis of CRC patients enrolled from the TCGA via the UCSC Xena database validated these relationships (r = 0.68 for *PLK4* and *SPDL1*, r = 0.70 for *KIF18A* and *SPDL1*, both *p* < 0.0001). 

The Reactome Pathway and KEGG BRITE databases were then applied to identify the *SPDL1*-related pathways and functional hierarchies, respectively, possibly implicated in colorectal cancer. The Reactome Pathway hierarchy panel for *SPDL1* and neighboring genes is depicted in Figure 4B, while the bar graph and heatmap showing the enrichment scores [−log10 (*p*-value)] or target genes, respectively, of the top 30 Reactome pathways are presented in Figure 4C,D. A complete listing of all significantly enriched pathways is presented in Table S6. The enriched functional terms related to the queried genes mainly included the cell cycle, mitotic events, cell cycle checkpoints, RHO GTPase signaling, retrograde vesicle-mediated transport from the Golgi to the endoplasmic reticulum, transcriptional regulation by *TP53*, DNA replication, and defective DNA repair (Figure 4C,D). More precisely, *SPDL1*-correlated gene set contained genes involved in chromosome condensation (e.g., *CCNB1*, *NCAPG*, *SMC4*, *NCAPH*), centriole duplication (e.g., *PLK4*, *NEK2*, *AURKA*), spindle assembly (e.g., *KIF18A*, *TTK*, *KIF11*, *KNSTRN*), kinetochore assembly/kinetochore microtubule dynamics and attachment stability (e.g., *CENPA*, *CENPE*, *CENPO*, *SGO2*), sister chromatid cohesion (e.g., *SGO1*, *SGO2*, *PTTG1*), chromosome segregation (e.g., *SGO1*, *CENPE*, *KIF11*, *KNSTRN*), spindle assembly checkpoint (e.g., *BUB1*, *BUBR1*, *CENPE*, *CENPO*, *KIF18A*, *ZWILCH*, *ZWINT*), APC-dependent catabolic process (e.g., *CCNA2*, *CCNB1*, *BUB1B*, *FBXO5*, *BUB1*), transcriptional regulation by *TP53* (e.g., *FANCI*, *CCNA2*, *CCNB1*, *EXO1*, *E2F7*), DNA replication (e.g., *CDC25A*, *TIMELESS*, *CCNA2*, *MCM10*, *CDC7*, *DNA2*), DNA replication checkpoint (e.g., *ATRIP*, *TOPBP1*, *MDC1*), replication stress response (e.g., *DNA2*, *MCM10*, *CDC7*, *TOP2A*), defective base excision repair (*NEIL3*), defective homology directed repair through homologous recombination (*EXO1*, *DNA2*), and others (Figure 4, Table S6). 

Consistently, KEGG Brite enrichment studies identified these genes to be predominantly chromosome and associated proteins, enzymes, DNA replication proteins, protein kinases, DNA repair and recombination proteins, membrane trafficking proteins, and cytoskeleton proteins (Figure 4E,F). 

**Figure 5:**
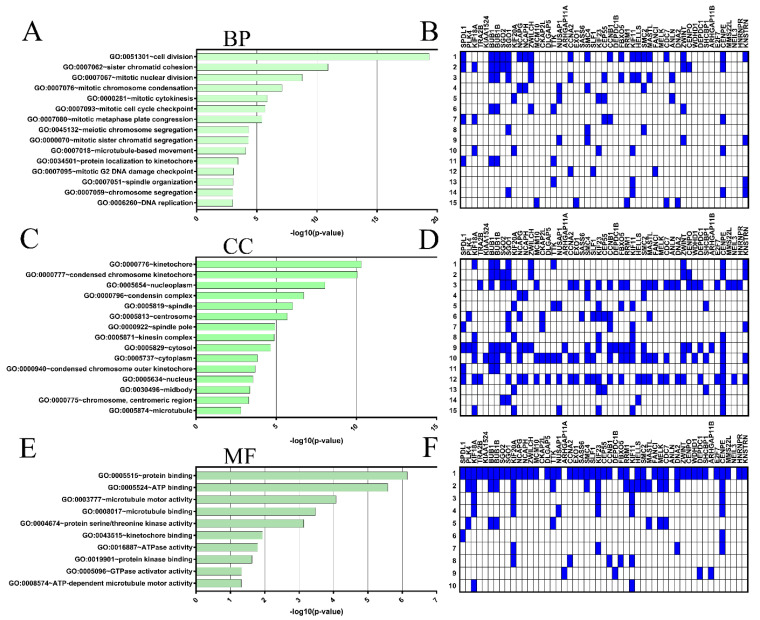
Gene ontology (GO) enrichment analysis for the top 50 genes positively correlated with *SPDL1* in CRC. The three GO categories [cellular component (CC), biological process (BP) and molecular function MF)] were detected using DAVID Bioinformatics Tool. The top 15 enriched terms for BP (**A**) and CC (**C**), as well as 10 terms for MF (**E**) are displayed. (**A**,**C**,**E**) The horizontal axis of bar chart represents the –log10 (*p*-value) for the GO terms represented on the vertical axis. (**B**,**D**,**F**) Heatmaps based on the target genes.

Next, GO function enrichment was performed with *SPDL1* and the top 50 co-expressed genes using the DAVID tool to analyze their possible activities in biological processes, molecular functions, and cellular components. The GO enrichment analysis indicated that our input gene set was significantly involved in 31 GO terms for biological processes (BP), 25 GO terms for cellular components (CC), and 10 GO terms for molecular functions (M); see Supplementary Table S7. The top 15 enrichment terms based on [−log10 (*p*-value)] (Figure 5A,C,E) and genes involved (Figure 5B,D,F) for BP and CC, as well as 10 terms for MF are shown in Figure 5 In this analysis, the most enriched ontology terms for *SPDL1* and co-expressed partners were cell division (GO: 0051301; Figure 5A), kinetochore (GO: 0000776; Figure 5B), and protein binding (GO: 0005515; Figure 5C).

## 3. Discussion

The tumor, lymph node, metastasis (TNM) staging system remains the main determinant of clinical outcome after surgical resection of colorectal cancer, and guides treatment decisions. However, there is substantial stage-independent variability in patients’ prognosis that is likely due to molecular heterogeneity. This variability drives the need for novel, robust prognostic and predictive biomarkers to inform treatment decisions including the use of adjuvant chemotherapy [13]. Given that genomic instability acts as a fuel of intertumor and intratumor genetic heterogeneity [14] and is a crucial feature in CRC development [13], molecular evidence from GIN and abnormal gene/protein expression has recently emerged as a rich resource to identify biomarkers for CRC [3]. In the present study, we therefore focused on a candidate protein–SPDL1, being suspected to underlie genomic (in-)stability in humans, and hypothesized that in CRC, there is a biological interdependence of the SPDL1 expression, clinical outcome, and GIN.

This hypothesis was put forward in the previous reports, including our recent one linking high levels of SPDL1 with genomic instability in pancreatic ductal adenocarcinoma [7] and oral squamous cell carcinoma [8]. Moreover, Kodama and coworkers termed SPDL1 a candidate CRC tumor suppressor, primarily based on functional studies in CRC cell lines and their xenograft models in nude mice with SPDL1 manipulation, as well as the adverse survival of *SPDL1* (mRNA)–low expressing CRC patients (TCGA data from GEPIA; *n* = 109) [10]. The latter observation is consistent with our TCGA-based survival analysis showing that *SPDL1*-low expression levels confer inferior OS in CRC. We further adjusted our estimates for confounder bias, and found that *SPDL1*-low mRNA expression was a negative prognostic factor in colorectal cancer independently of age at diagnosis, gender, and TNM parameters stage. Notably, stratification by *SPDL1* expression within two subgroups of different tumor stage (stage II and stage III–IV) identified patients with significantly different OS within the same stage subgroup. Furthermore, to our best knowledge, the current study is designed first to validate these results in clinical CRC specimens at the protein level. The comparison with adjacent normal tissue revealed an up-regulation of SPDL1 protein in a subset of tumor samples (48.84%), and these had better prognosis than the SPDL1-low expression counterpart even after adjusting for multiple confounders. Adjustment for PNI and LVI was not performed due to large amounts of missing data (as recommended by REMARK criteria [15]). We acknowledge this missingness as a limitation of our study and emphasize that the adjustment for either of these variables may have attenuated the significance of the results. However, our multivariate model still incorporated clinicopathological features such as, e.g., pT, pN, and pM, remaining the most important determinants of prognosis in CRC and being the basis of all authoritative patient management guidelines [16]. Again, our results for AJCC stage subgroups were comparable to the overall cohort, meaning that the positive impact of SPDL1-high expression on OS held up within stage II-III and stage IV tumors. However, in the TMA cohort, these subgroup analyses suffered from the limitation of small sample size and there was insufficient statistical power to reach statistical significance. Furthermore, although the multiple imputation method was adopted to adjust for the bias of missing data (according to REMARK criteria [15]), it could neither provide an unbiased estimation nor completely remove potential confounding. Therefore, we presented the results of survival analysis obtained with imputation alongside those from complete case analysis, and found that SPDL1 was still able to predict clinical outcome both in the univariate and multivariate analyses. In the subsequent survival analysis, we made distinction between low and moderate expression levels of SPDL1, and demonstrated that it was the latter expression status that gave the worst median OS compared to low and high expression levels. Importantly, SPDL1 expression retained prognostic power as a continuous variable, which seems important for increasing the likelihood of the reproducibility of our study. 

The association of SPDL1-high expression with superior survival is in line with tumor suppressive character, nevertheless our study does not allow us to infer anything conclusively about the nature of SPDL1 functionality in colorectal carcinogenesis, since showing that a biomarker is predictive of prognosis is merely a description of correlation and suggests, but does not robustly acknowledge, a biologic mechanism of functional significance [17]. Furthermore, we did not detect any marked differences in clinicopathological features of patients whose tumors exhibited SPDL1 overexpression compared to the remainder of the cohort, the presence of which would be expected with the protein acting as a tumor suppressor; though tumors with SPDL1-low expression tended to show more frequent perineural invasion; however, the difference was not statistically significant. In our cohort, this analysis was however hampered due to the specific distribution of the clinical and pathological data, especially the lack of well differentiated (G1) and early invasive (pT1) tumors, as well as the high number of missing data for PNI. Future studies involving larger cohort sizes with complete data equally balanced with regard to grade and stage may detect potential differentiating clinicopathological features. It is also uncommon to find overexpression of tumor suppressor in cancer tissue, yet SPDL1 expression as continuous and categorical variable was significantly elevated in CRC tissues compared to the control both at the protein and mRNA levels. However, this still does not rule out a possible tumor suppressor role of this protein in CRC since–though rarely and intriguingly, overexpression of some known tumor suppressors, e.g., p16Ink4a, has been described in several tumors [18]. It may be the case that this phenomenon is indicative of an attempt to stop tumor cell proliferation, e.g., via the induction of cell senescence [18]. Although the latest studies on SPDL1 missense variant in relation to the pathology of idiopathic pulmonary fibrosis (IPF) [19,20] and cancer protection [19] have suggested the association of SPDL1 with cell senescence, our bioinformatic analyses did not confirm this result, at least with regard to CRC, as we found *SPDL1* to be positively correlated with, e.g., *MKI67* (coding for marker of cell proliferation, Ki-67) and *TRA2B*, *KIAA1524*, *PRC1*, *SKP2* (coding for anti-senescence factors [21,22,23]), but not with the key senescence promoting genes. Thus, in the present considerations, it cannot be ignored that classically, the primary functions attributed to SPDL1 have been SAC silencing as well as regulation of chromosome alignment, kinetochore compaction and microtubule attachment to kinetochores during prometaphase, all of which contribute to a positive regulation of mitotic progression [24,25,26,27]. It is therefore tempting to speculate that one possible mechanism by which SPDL1 halts colorectal cancer progression (or more precisely–confers better patient prognosis) may potentially involve a severe potentiation of CIN upon its overexpression, as occurs with other proteins required for faithful mitosis [28]. Indeed, CIN has been shown to be poorly tolerated by cancer cells when induced through spindle assembly checkpoint inactivation or multipolar mitoses [29,30]. Notably, Kops et al. indicated that a checkpoint response was essential for cell survival, including that of chromosomally instable colorectal cancer cells [29]. Pursuing this assumption in the analysis of public datasets, we found that *SPDL1* expression in CRC was indeed markedly linked to genome instability phenotype, including CIN/aneuploidy score. Moreover, it positively coincided with the expression of DNA mismatch repair (MMR) genes, and as mentioned above with the *MKI67* gene, which seems consistent with the postulation that high-level expression of MMR molecules parallels features of genomic instability and occurs hand-in-hand with the proliferation activity of tumor cells [31,32,33]. *SPDL1* was also positively associated with *FOXM1* expression, which is an important predictor of genome instability [4], as well as with other genes of the CIN25 signature (with the exception of *TP53* gene) whose expression is consistently correlated with total functional aneuploidy in solid tumors [12]. As expected, analyses of biological functions of gene co-expression set around *SPDL1* revealed that the predicted signaling pathways, functional hierarchies, and gene ontological features spanned a broad spectrum of activities involved in genome maintenance and cell proliferation, the dysregulation of which may cause or indicate GIN. In addition to genes critically involved in the cell cycle and mitosis, *SPDL1*-correlated gene set contained checkpoint members of both chromosome segregation and DNA replication, as well as those associated with defective DNA repair or retrograde Golgi-to-ER trafficking, the latter of which has relatively recently become implicated in sustaining an integral genome [34,35]. Hence, wet-laboratory studies on SPDL1 and correlated pathways in CRC are warranted, as they may provide some novel insights into the mechanism of genomic instability in colorectal cancer. 

The results of the present study are in agreement with our recent data on pancreatic ductal adenocarcinoma [7]; however, opposite findings have been made in oral squamous cell carcinoma, where SPDL1 overexpression was reported to be related to CIN and worse outcome [8]. This may reflect the tumor tissue-specific role of SPDL1 and/or stage-specific, tumoral genetic background-specific or severity-dependent differences in the prognostic impact of genomic instability among various cancers. It is simultaneously clear that due to very little available information regarding SPDL1 in human cancers, many aspects of its function and regulation are still unresolved. Recent findings implicated SPDL1 in cell migration, and this activity may be also related to its overexpression in CRC. Indeed, the above cited in vitro and animal model study by Kodoma et al. has suggested that SPDL1′s tumor suppressor role in CRC is exerted through its effect on cell invasion and migration, as SPDL1-depleted cells showed significantly increased invasion and migration compared with control cells [10]. This is in contrast to human osteosarcoma U2OS cells and primary fibroblasts lacking SPDL1, which migrated slower in 2D cell culture than control cells [6]. Further studies are therefore required to place the prognostic relevance of SPDL1 in a cohesive biological context. From our results, we can more confidently conclude that SPDL1 fits well the feature of a double-faced protein, the level of which needs to be tightly regulated in tumors: moderate expression levels associate with a particularly poor prognosis in CRC, while high expression levels with improved prognosis. 

## 4. Materials and Methods

### 4.1. Patients and Tissue Material

The study was conducted on tumor and adjacent non-tumor tissue samples collected from patients undergoing colectomy in 2010–2017 at the Department of General, Hepatobiliary and Transplant Surgery of Dr A. Jurasz from the University Hospital No. 1 in Bydgoszcz. Patients diagnosed with colorectal adenocarcinoma were included in this investigation, while the exclusion criteria were as follows: hereditary colorectal cancer, neoadjuvant chemotherapy before resection, and reoperation due to recurrent cancer. The study group included 86 patients (37 females, 49 males) with an average age of 67 years at operation (median 67, range 41–100). Herein, pathologic TNM stage is based on the American Joint Committee on Cancer (AJCC) 8th edition. The AJCC TNM stage distribution is stage I, 5 (5.81%), stage II, 19 (22.09%), stage III, 21 (24.42%), and stage IV, 41 (47.67%). Due to postoperative death occurring within 30 days after surgery, 11 patients were excluded from survival analyses. Median overall survival and follow-up time in the investigated group amounted to 39.1 months, 95% CI 30.5–47.6 and 69.7 months, respectively. Histologically normal tissues adjacent to the tumor were available for 27 cases, and these constituted the control group. The same cohort of patients was previously described [36]. This study was carried out in accordance with the guidelines of the Declaration of Helsinki. The protocol was approved by the Nicolaus Copernicus University Ethics Committee (no. 337/2018).

### 4.2. Tissue Microarrays and Immunohistochemical Staining 

Immunohistochemical (IHC) staining was performed on tissue microarrays, the construction of which was described in our previous reports [36,37]. SPDL1 protein was stained in BenchMark^®^ ULTRA (Roche Diagnostics/Ventana Medical Systems, Tucson, AZ, USA) using ultraView Universal DAB Detection Kit (Roche Diagnostics/Ventana, Tucson, AZ, USA) according to the previously validated protocol [7]. Tissue sections were incubated with primary rabbit polyclonal anti-SPDL1 (1:100, PA5-60797, Thermo Fisher Scientific, Waltham, MA, USA) antibodies for 32 min at RT. Sections of normal human testis were used as a positive control, while a negative control was obtained by omitting the primary antibody. 

### 4.3. Evaluation of Immunohistochemical Staining

The protein expression was evaluated at 20x original objective magnification in a blinded fashion by two independent researchers (DG, AKW), including one senior pathologist (DG) using the light ECLIPSE E400 microscope (Nikon Instruments Europe, Amsterdam, The Netherlands). All disagreements regarding SPDL1 staining were resolved by a consensus meeting. Immunoexpression of the examined protein was scored according to the modified Remmele-Stegner Index (IRS) being the product of the percentage of positively stained cells/areas (0–4) and staining intensity (0–3). The percentage of positive cells/area (PS) was evaluated as follows: (0) less than 10% of stained cells/area, (1) 11–20% of stained cells/area, (2) 21–50% of stained cells/area, (3) 51–80% of stained cell/area and (4) equal or more than 81% of stained cells/area. In turn, staining intensity (IS) was measured on a 4-point scale as (0) negative, (1) weak staining, (2) moderate staining, and (3) strong staining. To determine “high” or “low” expression levels of SPDL1, the final IRS scores were dichotomized based on the optimal cut-off point established with the cutp function of the Evaluate Cutpoints software [38]. The cut-point values for high and low expression groups were <7.5; ≥7.5, respectively.

### 4.4. Publicly Available Transcriptomic Data

RNA sequencing (RNA-seq)-based mRNA expression data along with clinicopathological data for 275 colorectal cancer cases and 297 normal colorectal tissues were collected from The Cancer Genome Atlas (TCGA) and Genotype-Tissue Expression (GTEx) through UCSC Xena Browser (http://xena.ucsc.edu/, accessed on 22 June 2021) and www.cBioPortal.org (accessed on 22 June 2021). Stage I, II, III, and IV comprised 16.36% (*n* = 45), 38.91% (*n* = 107), 30.91% (*n* = 85), and 13.82% (*n* = 38), respectively. *SPDL1* expression levels were normalized by DESeq2 normalization, and then dichotomized into low-level and high-level groups according to the optimal cut-off point determined with the cutp function of the Evaluate Cutpoints software [38]. The cut-point values for low and high expression levels of *SPDL1* were <9.992; ≥9.992, respectively. Median overall survival and follow-up in the TCGA cohort amounted to 83.2 months, 95% CI 54.5–112.0 and 26.5 months, respectively. 

### 4.5. Functional Enrichment Analysis

Genes that were co-expressed with *SPDL1* in colon cancer tissues were predicted using the UALCAN database (http://ualcan.path.uab.edu/, accessed on 14 September 2021) [39], and the top 50 co-upregulated genes were subjected for further analyses. The verification of the expression profiles of these genes in colon and rectal cancer tissues relative to the respective control tissues was performed using the public web server GEPIA (Gene Expression Profiling Interactive Analysis; http://gepia.cancer-pku.cn/, accessed on 18 September 2021). Pathway analysis and visualization were conducted using the Reactome Pathway database (https://reactome.org, accessed on 18 September 2021) [40]. Gene ontology (GO) enrichment analysis was performed via the Database for Annotation, Visualization and Integrated Discovery version 6.8 (DAVID; https://david.ncifcrf.gov, accessed on 18 September 2021) to examine the ontology terms over-represented among the queried genes. Functional enrichment was performed in 3 categories of GO terms: biological process (BP), molecular function (MF) and cellular component (CC). The KEGG BRITE (https://www.genome.jp/kegg/brite.html, accessed on 14 September 2021) was utilized to explore functional hierarchies of the imputed genes. 

### 4.6. Statistical Analysis 

Statistical analyses were performed with the GraphPad Prism (version 8.0, GraphPad Software, San Diego, CA, USA) and SPSS software packages (version 26.0, IBM Corporation, Armonk, NY, USA). The normality of the data was evaluated using the Shapiro–Wilk test. The Mann–Whitney test was used to compare continuous variables. The strength and direction of the relationship between continuous variables were measured by Pearson or Spearman correlation coefficient (r). The two-tailed Chi-squared test or Fisher’s exact test was performed to evaluate differences between SPDL1 expression status and clinicopathological characteristics in CRC patients. Kaplan-Meier overall survival (OS) analysis was used to create survival curves, which were compared by log-rank test. Proportional hazard assumption was verified by testing for significant interactions when each variable was entered as a time-based covariate. Univariate and multivariate Cox proportional hazards regression analyses were performed to estimate the crude hazard ratios (HRs), adjusted HRs and 95% CI of HRs. Adjustment variables included age at diagnosis (≤65 vs. >65 years), gender (male vs. female), tumor grade (TMA cohort: intermediate vs. high; TCGA cohort: low and intermediate vs. high), pT (T1–T2 vs. T3–T4), pN (N0 vs. N1–N2), pM (absent vs. present), primary tumor location (rectum [ref.] vs. right or left), resection margin (R0 vs. R1−R2). Separate multivariate Cox models were built with the overall AJCC TNM stage (TCGA cohort: stage I [ref.] vs. II or III or IV; TMA cohort: stage IV [ref.] vs. I or II or III). Two independent survival analyses were performed: categorical and continuous data analysis. For categorization, which is clinically desired for issues such as decision making [15], we applied outcome driven cutpoint analysis based on the optimal dichotomy for survival difference. Analogous survival analyses were performed when immunoreactive scores were treated as a continuous variable to demonstrate dose-effects of the biomarker expression and increase the reproducibility of the study. Multiple imputation (MI) of missing covariates for the Cox proportional hazards model was used to adjust for the bias of missing data (Table S8), but the variables with amounts of missing data over 20% (PNI, VI) were not considered for the model. It was assumed that the missing data of predictor variables occurred at random. Missing data in evaluated variables were multiple-imputed and combined estimates were obtained from 5 imputed datasets. Statistical significance was assumed as two-sided *p* < 0.05.

## 5. Conclusions

In summary, our results supported the notion that altered SPDL1 expression in CRC tissue might predict patient outcome, and mechanistically this was likely attributable, at least partially, to tipping the balance of chromosomal instability toward the detriment of tumor overall longevity. For eventual clinical application, future independent validation studies and further wet lab-based investigations are needed.

## Figures and Tables

**Table 1:** Association of SPDL1 protein expression with clinicopathological parameters of colorectal cancer patients.

Clinicopathological Feature	*n* (%) *n* = 86	SPDL1 Expression		*p* Value
		Low *n* = 44	High *n* = 42	
Age (years)				
≤65	38 (44.19)	20 (52.63)	18 (47.37)	0.83
>65	48 (55.81)	24 (50.00)	24 (50.00)	
Gender				
Male	49 (56.98)	23 (46.94)	26 (53.06)	0.39
Female	37 (43.02)	21 (56.76)	16 (43.24)	
Grading				
G2	76 (91.57)	39 (51.32)	37 (48.68)	0.71
G3	7 (8.43)	3 (42.86)	4 (57.14)	
pT status				
T2	13 (15.12)	6 (46.15)	7 (53.85)	0.70
T3	60 (69.77)	30 (50.00)	30 (50.00)	
T4	13 (15.12)	8 (61.54)	5 (38.46)	
pN status				
N0	33 (40.74)	16 (48.48)	17 (51.52)	0.66
N1–N2	48 (59.26)	26 (54.17)	22 (45.83)	
pM status				
M0	42 (52.50)	20 (47.62)	22 (52.38)	0.51
M1	38 (47.50)	21 (55.26)	17 (44.74)	
TNM stage				
I	5 (6.25)	2 (40.00)	3 (60.00)	0.31
II	17 (21.25)	7 (41.18)	10 (58.82)	
III	20 (25.00)	11 (55.00)	9 (45.00)	
IV	38 (47.50)	21 (55.26)	17 (44.74)	
VI				
Absent	24 (60.00)	10 (41.67)	14 (58.33)	0.52
Present	16 (40.00)	9 (56.25)	7 (43.75)	
PNI				
Absent	25 (89.29)	10 (40.00)	15 (60.00)	0.09
Present	3 (10.71)	3 (100.00)	0 (0.00)	
Resection margin				
R0	65 (75.58)	33 (50.77)	32 (49.23)	>0.99
R1−R2	21 (24.42)	11 (52.38)	10 (47.62)	
Primary tumor location				
Right colon	30 (34.88)	16 (53.33)	14 (46.67)	0.54
Left colon	28 (32.56)	12 (42.86)	16 (57.14)	
Rectum	28 (32.56)	16 (57.14)	12 (42.86)	

Abbreviations: VI—vascular invasion, PNI—perineural invasion. TNM stage is based on AJCC 8th edition.

**Table 2:** Univariate and multivariate Cox regression analyses of OS for CRC patients in our cohort (*n* = 75).

Variable	Univariate Analysis				Multivariate Analysis			
	HR	95% CI		*p*	HR	95% CI		*p*
		Lower	Upper			Lower	Upper	
SPDL1	0.45	0.25	0.82	**0.009**	0.42	0.23	0.78	**0.006**
age	1.09	0.61	1.93	0.77	1.42	0.73	2.74	0.30
gender	0.99	0.55	1.75	0.96	1.32	0.71	2.45	0.39
grade	2.58	1.00	6.69	0.05	3.87	1.36	11.01	**0.01**
pT	2.30	0.91	5.83	0.08	1.88	0.72	4.93	0.20
pN	1.70	0.93	3.11	0.09	1.16	0.58	2.29	0.68
pM	2.81	1.55	5.08	**0.001**	3.32	1.73	6.37	**0.0003**
TNM stage	2.93	1.37	6.27	**0.006**	-	-	-	-
resection margin	1.86	0.98	3.53	0.06	2.37	1.13	4.98	**0.02**
tumor location								
rectum	Ref.				Ref.			
right colon	1.29	0.66	2.52	0.46	2.10	1.00	4.44	0.05
left colon	0.99	0.49	2.01	0.98	1.55	0.71	3.40	0.28

Abbreviations: CI—confidence interval, CRC—colorectal cancer, HR—hazard ratio, OS—overall survival, pT—primary tumor, pN-regional lymph node, pM—distant metastasis, Ref.—reference. TNM stage is based on AJCC 8th edition. AJCC TNM stage categories are: I–II vs. III–IV. Significant *p*-values (*p* < 0.05) are indicated in bold.

**Table 3:** Association of *SPDL1* mRNA expression with clinicopathological parameters of colorectal cancer patients.

Clinicopathological Feature	*n* (%) *n* = 275	*SPDL1* Expression		*p* Value
		Low *n* = 234	High *n* = 41	
Age (years)				
≤65	129 (46.91)	106 (82.17)	23 (17.83)	0.42
>65	146 (53.09)	128 (87.67)	18 (12.33)	
Gender				
Male	150 (54.55	125 (83.33)	25 (16.67)	0.40
Female	125 (45.45)	109 (87.20)	16 (12.80)	
pT status				
T1	6 (2.18)	6 (100.00)	0 (0.00)	0.67
T2	43 (15.64)	39 (90.70)	4 (9.30)	
T3	188 (68.36)	154 (81.91)	34 (18.09)	
T4	38 (13.82)	35 (92.11)	3 (7.89)	
pN status				
N0	160 (58.18)	137 (85.63)	23 (14.38)	0.86
N1–N2	115 (41.82)	97 (84.35)	18 (15.65)	
pM status				
M0	232 (86.25)	196 (84.48)	36 (15.52)	>0.99
M1	37 (13.75)	32 (86.49)	5 (13.51)	
TNM stage				
I	44 (16.36)	40 (90.91)	4 (9.09)	0.60
II	106 (39.41)	88 (83.02)	18 (16.98)	
III	82 (30.48)	67 (82.72)	14 (17.28)	
IV	37 (13.75)	32 (86.49)	5 (13.51)	

**Table 4:** Univariate and multivariate Cox regression analyses of OS for CRC patients in TCGA cohort (*n* = 275).

Variable	Univariate Analysis				Multivariate Analysis			
	HR	95% CI		*p*	HR	95% CI		*p*
		Lower	Upper			Lower	Upper	
*SPDL1*	0.41	0.18	0.96	**0.04**	0.37	0.16	0.85	**0.02**
age	1.68	1.01	2.81	**0.047**	2.25	1.30	3.88	**0.004**
gender	1.42	0.86	2.33	0.17	1.13	0.68	1.88	0.65
pT	3.23	1.17	8.89	**0.02**	2.23	0.78	6.39	0.14
pN	2.45	1.50	4.02	**0.0004**	1.90	1.09	3.31	**0.02**
pM	3.71	2.16	6.36	**<0.0001**	3.23	1.75	5.94	**0.0002**
TNM stage	2.97	1.78	4.96	**<0.0001**	-	-	-	-

AJCC TNM stage categories are: I–II vs. III–IV; abbreviations: CI—confidence interval, CRC—colorectal cancer, HR—hazard ratio, pT—primary tumor, pN—regional lymph node, pM—distant metastasis, OS—overall survival, TCGA—The Cancer Genome Atlas. “-“ indicates variable was not included in multivariate Cox analysis. Significant *p*-values (*p* < 0.05) are indicated in bold.

## Data Availability

Publicly available datasets were analyzed in this study. These data can be found here: http://www.cbioportal.org/study/summary?id=paad_tcga_pan_can_atlas_2018 (accessed on 22 June 2021); https://xenabrowser.net (accessed on 22 June 2021), http://ualcan.path.uab.edu/ (accessed on 14 September 2021); GEPIA, Gene Expression Profiling Interactive Analysis (http://gepia.cancer-pku.cn/, accessed on 18 September 2021); DAVID, Database for Annotation, Visualization and Integrated Discovery version 6.8 (https://david.ncifcrf.gov, accessed on 18 September 2021); KEGG BRITE (https://www.genome.jp/kegg/brite.html, accessed on 14 September 2021). Our own data presented in this study are available on request from the corresponding author. The data are not publicly available due to ethical restrictions.

## Supplementary Materials

The following supporting information can be downloaded at: https://www.mdpi.com/article/10.3390/ijms23031819/s1.
