# Supplementary material for: SPDL1 Is an Independent Predictor of Patient Outcome in Colorectal Cancer
Source: Int J Mol Sci. 2022 Feb 5;23(3):1819. doi: 10.3390/ijms23031819 (PMC8836361; doi:10.3390/ijms23031819)
Supplement: Supplementary file 1 [file ijms-23-01819-s001.zip › ijms-1541328-supplementary.pdf]

International Journal of Molecular Sciences

MDPI

Table S1. Univariate and multivariate Cox regression analyses of OS for CRC patients in the TMA cohort (n=75) with the AJCC TNM stages as the adjusting variables instead of pT, pN, pM categories.

Variable	Univariate analysis				Multivariate analysis			
	HR	95% CI		*p*	HR	95% CI		*p*
		lower	upper			lower	upper	
SPDL1	0.45	0.25	0.82	**0.009**	0.42	0.22	0.79	**0.008**
age	1.09	0.61	1.93	0.77	1.40	0.72	2.71	0.32
gender	0.99	0.55	1.75	0.96	1.34	0.72	2.49	0.35
grade	2.58	1.00	6.69	0.05	4.28	1.51	12.14	**0.006**
pT	2.30	0.91	5.83	0.08	-	-	-	-
pN	1.70	0.93	3.11	0.09	-	-	-	-
pM	2.81	1.55	5.08	**0.001**	-	-	-	-
resection margin	1.86	0.98	3.53	0.06	2.28	1.06	4.89	**0.04**
tumor stage								
stage IV		Ref.				Ref.		
stage I	0.18	0.02	1.31	0.09	0.18	0.02	1.39	0.10
stage II	0.28	0.12	0.64	**0.003**	0.27	0.11	0.65	**0.004**
stage III	0.48	0.24	0.98	**0.04**	0.31	0.14	0.68	**0.003**
tumor location								
rectum		Ref.				Ref.		
right colon	1.29	0.66	2.52	0.46	2.19	1.06	4.49	**0.03**
left colon	0.99	0.49	2.01	0.98	1.56	0.71	3.44	0.27

Abbreviations: CI - confidence interval, CRC - colorectal cancer, HR - hazard ratio, OS - overall survival, pM - distant metastasis, pN - regional lymph node, pT - primary tumor, Ref. - reference, TMA - tissue microarray. TNM stage is based on AJCC 8th edition. Significant p-values ($p < 0.05$) are indicated in bold.

Table. S2. Univariate and multivariate Cox regression analyses of OS for CRC patients in the TMA cohort (n=75). Expression of SPDL1 was used as a continuous variable.

Variable	Univariate analysis				Multivariate analysis: SPDL1 (IRS)				Multivariate analysis: SPDL1 (IS)			
	HR	95% CI		*p*	HR	95% CI		*p*	HR	95% CI		*p*
		lower	upper			lower	upper			lower	upper	
SPDL1 (IRS)	0.86	0.75	0.99	**0.03**	0.82	0.70	0.96	**0.01**	-	-	-	-
SPDL1 (IS)	0.55	0.32	0.94	**0.03**	-	-	-	-	0.45	0.24	0.85	**0.01**
age	1.00	0.97	1.03	0.86	1.49	0.76	2.92	0.24	1.48	0.76	2.90	0.25
gender	0.99	0.55	1.75	0.96	1.34	0.72	2.50	0.36	1.32	0.71	2.46	0.38
grade	2.58	1.00	6.69	0.05	3.79	1.32	10.88	**0.01**	3.78	1.32	10.85	**0.01**
pT	2.30	0.91	5.83	0.08	2.27	0.86	6.01	0.10	2.25	0.85	5.97	0.10
pN	1.70	0.93	3.11	0.09	1.05	0.53	2.07	0.88	1.04	0.53	2.05	0.92
pM	2.81	1.55	5.08	**0.001**	3.15	1.65	6.03	**0.001**	3.17	1.66	6.08	**0.0005**
TNM stage	2.93	1.37	6.27	**0.006**	**-**	**-**	**-**	**-**	**-**	**-**	**-**	**-**
resection margin	1.86	0.98	3.53	0.06	2.73	1.24	6.03	**0.01**	2.71	1.23	5.97	**0.01**
tumor location												
rectum		Ref.				Ref.				Ref.		
right colon	1.29	0.66	2.52	0.46	2.01	0.97	4.15	0.06	1.99	0.96	4.11	0.06
left colon	0.99	0.49	2.01	0.98	1.40	0.64	3.06	0.41	1.40	0.64	3.06	0.40

Abbreviations: CI - confidence interval, CRC - colorectal cancer, HR - hazard ratio, IRS - immunoreactive score, IS - staining intensity, OS - overall survival, pM - distant metastasis, pN - regional lymph node, pT - primary tumor.

TMA - tissue microarray. TNM stage is based on AJCC 8th edition. AJCC TNM stage categories are: I-II vs. III-IV. “-“ indicates variable was not included in multivariate Cox analysis. Significant p-values ($p < 0.05$) are indicated in bold.

Table. S3. Univariate and multivariate Cox regression analyses of OS for CRC patients in the TMA cohort. Analyses were restricted to individuals with complete data (n=59).

Variable	Univariate analysis				Multivariate analysis			
	HR	95% CI		*p*	HR	95% CI		*p*
		lower	upper			Lower	upper	
SPDL1	0.37	0.18	0.75	**0.006**	0.30	0.13	0.70	**0.005**
age	1.40	0.70	2.77	0.34	2.26	0.88	5.83	0.09
gender	0.81	0.41	1.61	0.55	1.32	0.60	2.91	0.49
grade	2.16	0.75	6.23	0.15	2.85	0.88	9.20	0.08
pT	1.73	0.61	4.94	0.30	1.26	0.40	4.01	0.69
pN	2.01	0.98	4.13	0.06	1.56	0.69	3.52	0.29
pM	3.05	1.51	6.19	**0.002**	2.39	1.06	5.36	**0.04**
TNM stage	3.76	1.45	9.74	**0.006**	-	-	-	-
resection margin	1.66	0.72	3.83	0.23	3.06	0.90	10.38	0.07
tumor location								
rectum		Ref.				Ref.		
right colon	1.41	0.62	3.22	0.42	2.51	0.96	6.57	0.06
left colon	1.20	0.49	2.95	0.70	2.86	0.90	9.13	0.08

Abbreviations: CI - confidence interval, CRC - colorectal cancer, HR - hazard ratio, OS - overall survival, pM - distant metastasis, pN - regional lymph node, pT - primary tumor, TMA - tissue microarray. TNM stage is based on AJCC 8th edition. AJCC TNM stage categories are: I-II vs. III-IV. “-“ indicates variable was not included in multivariate Cox analysis. Significant p-values ($p < 0.05$) are indicated in bold.

Table. S4. Univariate and multivariate Cox regression analyses of OS for CRC patients in the TCGA cohort (n=275) with the TNM stages as the adjusting variables instead of pT, pN, pM categories.

Variable	Univariate analysis				Multivariate analysis			
	HR	95% CI		*p*	HR	95% CI		*p*
		lower	upper			lower	upper	
SPDL1	0.41	0.18	0.96	**0.04**	0.38	0.16	0.88	**0.02**
age	1.68	1.01	2.81	**0.047**	2.25	1.30	3.89	**0.004**
gender	1.42	0.86	2.33	0.17	1.12	0.67	1.86	0.68
pT	3.23	1.17	8.89	**0.02**	-	-	-	-
pN	2.45	1.50	4.02	**0.0004**	-	-	-	-
pM	3.71	2.16	6.36	**<0.0001**	-	-	-	-
tumor stage								
stage I		Ref.				Ref.		
stage II	2.25	0.67	7.64	0.19	2.34	0.69	7.93	0.17
stage III	4.30	1.30	14.26	**0.02**	5.12	1.54	17.01	**0.008**
stage IV	10.08	2.98	34.14	**0.002**	14.47	4.20	49.81	**<0.0001**

Abbreviations: CI - confidence interval, CRC - colorectal cancer, HR - hazard ratio, OS - overall survival, pM - distant metastasis, pN - regional lymph node, pT - primary tumor, Ref. – reference. TNM stage is based on AJCC 8th edition. Significant p-values ($p < 0.05$) are indicated in bold.

Table S5. A medium-confidence (≥ 0.4) set of *SPDL1*-positively correlated genes.

Genes	R
PLK4	0.72
KIF18A	0.72
TRA2B	0.71
KIAA1524	0.70
BUB1	0.70
BUB1B	0.70
SGOL2	0.69
SGOL1	0.68
KIF20A	0.68
NCAPG	0.67
NCAPH	0.67
ZWILCH	0.67
MCM10	0.66
CKAP2L	0.66
DLGAP5	0.66
TTK	0.65
NUSAP1	0.65
ARHGAP11A	0.65
CCNA2	0.65
EXO1	0.65
SASS6	0.65
SMC4	0.65
ANKRD32	0.64
KIF23	0.64
CEP55	0.64
CCNB1	0.64
DEPDC1B	0.64
FBXO5	0.63
RRM1	0.63
KIF11	0.63
HELLS	0.63
SMC2	0.63
MASTL	0.63
FANCI	0.63
MELK	0.62
CDC7	0.62
ANLN	0.62
DNA2	0.62
ZWINT	0.62
CENPO	0.62
WDHD1	0.62
DEPDC1	0.62
SHCBP1	0.62
ARHGAP11B	0.61
E2F7	0.61
CENPE	0.61
C6orf167	0.61
NEIL3	0.61
HNRNPR	0.60
C15orf23	0.60
HJURP	0.60
CENPA	0.60
RFC5	0.60
MCM6	0.60
C10orf119	0.60
BRIP1	0.60
RFC4	0.59
RARS	0.59
GSTCD	0.59
PRC1	0.59
CDC23	0.59
HAT1	0.59
CDK1	0.59
EXOSC9	0.59
BUB3	0.59
ASPM	0.59
STIL	0.59
KIF2C	0.59
**Genes**	**R**
LMNB1	0.58
G3BP1	0.58
RACGAP1	0.58
CEP78	0.58
KIF14	0.58
ATAD5	0.58
KIF20B	0.58
CHEK1	0.58
POLQ	0.58
TIPIN	0.58
SKA2	0.58
CHAF1A	0.58
ECT2	0.58
NEDD1	0.58
DNMT1	0.58
HMMR	0.58
CDC45	0.57
MKI67	0.57
CLSPN	0.57
DTL	0.57
CASC5	0.57
FANCM	0.57
SFRS1	0.57
ORC1L	0.57
SPAG5	0.57
TOP2A	0.57
GSG2	0.57
LIN54	0.57
DNAJC9	0.56
CCNB2	0.56
SCLT1	0.56
WDR76	0.56
SFRS13A	0.56
CDC20	0.56
TAF5	0.56
KIF15	0.56
KIF18B	0.56
GINS3	0.56
HMGXB4	0.55
SFXN1	0.55
DONSON	0.55
GABPB1	0.55
MSH2	0.55
C18orf54	0.55
NEK2	0.55
PRR11	0.55
GTSE1	0.55
TOPBP1	0.55
UHRF1	0.55
CPSF6	0.55
PLK1	0.55
GRPEL2	0.54
FANCD2	0.54
NUF2	0.54
SPC25	0.54
HBS1L	0.54
CCAR1	0.54
CENPI	0.54
NCAPD3	0.54
IPO11	0.54
SFRS7	0.54
CASP8AP2	0.54
CDCA5	0.54
HNRNPA2B1	0.54
RAD51	0.54
SET	0.54
NDC80	0.54
TRIP13	0.54
**Genes**	**R**
EZH2	0.53
CTPS	0.53
CHAF1B	0.53
SUV39H2	0.53
E2F8	0.53
XPO1	0.53
WDR62	0.53
CDC27	0.53
NUP188	0.53
MAD2L1	0.53
MCM2	0.53
FEN1	0.53
USP1	0.53
MSH6	0.53
HOMER1	0.53
TMEM194A	0.53
GMPS	0.53
KIF4A	0.52
TMPO	0.52
NUP160	0.52
KNTC1	0.52
PTTG1	0.52
SUZ12	0.52
NUP107	0.52
FAM54A	0.52
POLA2	0.52
VRK1	0.52
CNOT6	0.52
MTHFD2	0.52
TARDBP	0.52
LIN9	0.52
KIFC1	0.52
SKA1	0.52
INCENP	0.52
EXOSC2	0.52
PSIP1	0.52
DIAPH3	0.52
DDX46	0.52
WHSC1	0.52
CDK2	0.52
C11orf82	0.52
NPM1	0.52
PSMC3IP	0.52
BARD1	0.52
SFRS3	0.52
TCP1	0.51
RRM2	0.51
CKAP5	0.51
CDKN3	0.51
C14orf145	0.51
WDR43	0.51
CCDC138	0.51
CENPK	0.51
CDCA8	0.51
DHX15	0.51
TIMELESS	0.51
HAUS8	0.51
NAA15	0.51
C15orf42	0.51
RAD23B	0.51
EIF2S1	0.51
SKP2	0.51
DHX9	0.51
FAM98B	0.51
RAD18	0.51
MTBP	0.51
EPT1	0.50
CENPL	0.50
**Genes**	**R**
CDCA2	0.50
ESPL1	0.50
BBS7	0.50
CHUK	0.50
WDR67	0.50
ORC6L	0.50
ARL13B	0.50
TCF19	0.50
OIP5	0.50
MCM4	0.50
CCNF	0.50
DCLRE1B	0.50
NAA50	0.50
ABCE1	0.50
NOC3L	0.50
SSRP1	0.50
RPAP3	0.50
HNRNPC	0.50
ZNF714	0.50
GTF2H3	0.50
G2E3	0.50
DHFR	0.50
HSPA14	0.50
HAUS6	0.50
C1orf135	0.50
TNPO1	0.50
CWC27	0.50
MPHOSPH9	0.49
GINS4	0.49
KRR1	0.49
FAM111B	0.49
MCM3	0.49
BCCIP	0.49
RBM27	0.49
PARP2	0.49
TMEM48	0.49
PDE12	0.49
SMC3	0.49
PRIM1	0.49
CLEC2D	0.49
XRCC2	0.49
UCHL5	0.49
DDX1	0.49
TCOF1	0.49
BLM	0.49
GPN3	0.49
FAF2	0.49
UBA6	0.49
FANCB	0.49
TUBB	0.49
SRP72	0.49
FAM72D	0.49
PSMD6	0.49
UTP15	0.49
RIF1	0.49
RAD54L	0.49
KPNB1	0.49
CENPF	0.49
DCLRE1A	0.49
TUBA1B	0.49
ATAD2	0.49
POLE2	0.49
MTRF1L	0.49
SKIV2L2	0.48
NEK4	0.48
NUP50	0.48
SFRS2	0.48
CDC25C	0.48
**Genes**	**R**
LMNB2	0.48
PPP5C	0.48
NUP210	0.48
RAD1	0.48
IARS	0.48
RMI1	0.48
BRCA1	0.48
CDCA4	0.48
HNRNPK	0.48
DNAJB11	0.48
CAND1	0.48
HSPA4	0.48
RIBC2	0.48
ZNF367	0.48
METAP2	0.48
PDHX	0.48
CDC25A	0.48
LRRC58	0.48
H2AFZ	0.48
HNRNPL	0.48
NSD1	0.48
C4orf46	0.48
PCGF6	0.48
NCAPG2	0.48
SPATA5	0.48
CCNE2	0.47
KPNA2	0.47
CSTF3	0.47
GTPBP4	0.47
TDG	0.47
KIF2A	0.47
UBE2T	0.47
ZNF143	0.47
CEP135	0.47
ASF1B	0.47
SNRNP27	0.47
HAUS2	0.47
PSRC1	0.47
DHX29	0.47
PMS1	0.47
MRPL19	0.47
CENPN	0.47
RBMX	0.47
ETF1	0.47
HIATL1	0.47
MLF1IP	0.47
GEN1	0.47
RSRC1	0.47

PPM1G	0.47
PRPF4	0.47
ANP32E	0.47
LEO1	0.47
USP13	0.47
PSMA4	0.47
PA2G4	0.47
UIMC1	0.46
NCL	0.46
SFPQ	0.46
RQCD1	0.46
HSPA9	0.46
C10orf18	0.46
RTKN2	0.46
DSCC1	0.46
MTHFD1	0.46
HNRNPD	0.46
LIG1	0.46
RNASEH1	0.46
TMTC3	0.46
**Genes**	**R**
C4orf21	0.46
CKAP2	0.46
ALG10	0.46
DYNC1LI1	0.46
TCERG1	0.46
HNRNPF	0.46
C6orf182	0.46
ISOC1	0.46
TROAP	0.46
C12orf48	0.46
TTF2	0.46
WDR36	0.46
KIF24	0.46
FBXO45	0.46
AURKB	0.46
PPP3R1	0.46
SLBP	0.46
RAD51C	0.46
DENR	0.46
CEP97	0.46
HNRNPA3	0.46
LYAR	0.46
TUBGCP4	0.46
HNRNPU	0.46
CPSF2	0.46
HNRPLL	0.46
LTV1	0.46
ZNF639	0.46
ILF3	0.46
HNRNPH1	0.45
DDX21	0.45
CNOT10	0.45
SKA3	0.45
TAF3	0.45
MATR3	0.45
HNRNPM	0.45
GMNN	0.45
CHORDC1	0.45
ATL2	0.45
FOXN2	0.45
POLD1	0.45
GEMIN5	0.45
RECQL	0.45
DBF4	0.45
MTX3	0.45
PPAT	0.45
PAICS	0.45
ERCC6L	0.45
NUP37	0.45
SENP1	0.45
PTPN11	0.45
NUP43	0.45
NASP	0.45
PRIM2	0.45
UBR7	0.45
RBM17	0.45
BDP1	0.45
USP39	0.45
STT3A	0.45
UBA5	0.45
PATL1	0.45
UMPS	0.45
RAD54B	0.45
PSMD2	0.45
NHLRC2	0.45
RFC1	0.45
STIP1	0.45
CUL2	0.45
CCT5	0.45
PPIL1	0.44
FAM161A	0.44
PPP4R2	0.44
HMGB2	0.44
FIGNL1	0.44
**Genes**	**R**
LOC727896	0.44
BCLAF1	0.44
NUP205	0.44
RFC3	0.44
CEP152	0.44
NUDT5	0.44
EIF2AK2	0.44
PPP2CA	0.44
MOBKL1B	0.44
PTBP1	0.44
UHRF1BP1	0.44
MND1	0.44
FAR1	0.44
ZCCHC9	0.44
FANCA	0.44
CENPP	0.44
BZW1	0.44
RNASEH2A	0.44
C2orf44	0.44
C1orf112	0.44
VRK2	0.44
PTCD2	0.44
C5orf34	0.44
PRPF40A	0.44
DCP2	0.44
CANX	0.44
CHCHD4	0.44
NUP155	0.44
YME1L1	0.44
HNRNPA3P1	0.44
AMD1	0.44
BRCA2	0.44
CCT2	0.44
BMS1	0.44
TACC3	0.44
RIOK2	0.44
C2orf3	0.44
ZNF207	0.44
CRY1	0.44
PPP1CC	0.44
PSMD14	0.44
EWSR1	0.44
SMC6	0.43
ERCC8	0.43
SLC31A1	0.43
IPPK	0.43
SMC1A	0.43
FAM116A	0.43
RBM15	0.43
BIRC5	0.43
POLD3	0.43
NBN	0.43
CKS1B	0.43
TBP	0.43
PDS5A	0.43
EED	0.43
C17orf53	0.43
C21orf45	0.43
RAN	0.43
IFT81	0.43
PPWD1	0.43
POLR3G	0.43
TYMS	0.43
EXOSC10	0.43
GTF3C4	0.43
NCAPD2	0.43
ESCO2	0.43
ARHGAP19	0.43
MRPS18C	0.43
PANK3	0.43
ZNF695	0.43
SFRS12	0.43
SLC30A5	0.43
CORO1C	0.43
**Genes**	**R**
KIAA0101	0.43
PGM2	0.43
LSM11	0.43
ZFP91	0.43
DR1	0.43
LLPH	0.43
DBR1	0.43
DCUN1D5	0.42
TRAIP	0.42
SOCS4	0.42
C1orf96	0.42
TUBGCP5	0.42
HAUS3	0.42
NUP93	0.42
SLC4A1AP	0.42
LRRC40	0.42
CDC5L	0.42
RCC1	0.42
PGAM4	0.42
CCT4	0.42
PHAX	0.42
WDR75	0.42
SRP19	0.42
MRPL3	0.42
TUBG1	0.42
KHSRP	0.42
MRS2	0.42
YWHAQ	0.42
MRPS9	0.42
NFXL1	0.42
IDE	0.42
C14orf106	0.42
PNPT1	0.42
FOXM1	0.42
AHCTF1	0.42
MDC1	0.42
POLR1B	0.42
C5orf24	0.42
SAMD8	0.42
XPOT	0.42
BUD13	0.42
PBK	0.42
PSMD11	0.42
POLR3A	0.42
STAG1	0.42
PWP1	0.42
ZNF519	0.42
PTPLAD1	0.42
MAP2K1	0.42
SAE1	0.42
ZNF37A	0.42
VTA1	0.42
RNGTT	0.42
WDR12	0.42
MRPL30	0.42
IDH3A	0.42
SLC30A6	0.42
NOLC1	0.42
BRIX1	0.41
IPO9	0.41
AGPS	0.41
CWC22	0.41
NUS1	0.41
SAAL1	0.41
GLE1	0.41
CCDC18	0.41
UBE2K	0.41
LIMS1	0.41
MAPK8	0.41
FAM72A	0.41
SMNDC1	0.41
CPSF7	0.41
SNRPD3	0.41
TIFA	0.41
**Genes**	**R**
FANCC	0.41
NOP58	0.41
TAF9	0.41
SUPT16H	0.41
QSOX2	0.41
AEBP2	0.41
IREB2	0.41
SMARCA5	0.41
SNRPA1	0.41
MARS	0.41
LIN52	0.41
RBM14	0.41
ICMT	0.41
SYNCRIP	0.41
FAM72B	0.41
ZW10	0.41
SMN2	0.41
DDX18	0.41
MCM5	0.41
RPE	0.41
FUBP3	0.41
ZRANB3	0.41
GFM2	0.41
C9orf80	0.41
TRIM59	0.41
EAF1	0.41
LOC441089	0.41
HSP90AA1	0.41
NAA25	0.41
PGM3	0.41
UBXN2A	0.41
FAM98A	0.41
USP14	0.41
CEP120	0.41
C18orf55	0.41
STXBP4	0.41
ARNTL2	0.41
ATG4C	0.41
QSER1	0.41
MNS1	0.41
CEBPZ	0.41
TFAM	0.41
RAD51AP1	0.41
R3HDM1	0.41
DIMT1L	0.40
EIF4E	0.40
USP15	0.40
PRMT5	0.40
PCNP	0.40
CDT1	0.40
CCDC75	0.40
PELO	0.40
POLR2D	0.40
ETAA1	0.40
CIT	0.40
TSR1	0.40
POLK	0.40
C6orf211	0.40
UCK2	0.40
C10orf46	0.40
YY1	0.40
PARP1	0.40
MYO19	0.40
APITD1	0.40
UBE2D2	0.40
TIAL1	0.40
PA2G4P4	0.40
EBNA1BP2	0.40
ALMS1	0.40
CTDSPL2	0.40
COMMD2	0.40
XRCC6	0.40
LARP1	0.40
CACYBP	0.40
**Genes**	**R**
MCM7	0.40
CLPX	0.40
TARS	0.40
EEF1E1	0.40
ZNF346	0.40
CCDC41	0.40
POLR2B	0.40
HDAC3	0.40
ARSK	0.40
LOC100128191	0.40
BRI3BP	0.40
TCHP	0.40
POC5	0.40
METTL10	0.40
C1orf163	0.40
RFWD3	0.40
ZNF326	0.40
DCP1A	0.40
ENOPH1	0.40
LOC221710	0.40
CSNK1G1	0.40
XRCC5	0.40
RBM25	0.40
CENPH	0.40

PRPS1	0.40
FIP1L1	0.40
SART3	0.40
HSPD1	0.40
OGFOD1	0.40
CXCL10	0.40
TRIM37	0.40
COL4A3BP	0.40
SRFBP1	0.40
TIMM17A	0.40
MPHOSPH10	0.40
ADAM17	0.40
SPC24	0.40
TAF2	0.40
HSPA5	0.40

Table S6. Reactome pathway enrichment analysis for the top 50 genes positively correlated with *SPDL1* in colorectal cancer.

PATHWAY NAME	PATHWAY ID	NO. OF GENES	P VALUE	GENES
Cell Cycle, Mitotic	R-HSA-69278	27	1,11E-16	PLK4;ZWILCH;NCAPG;BUB1B;CDC7;KIF23;MCM10;MASTL;SMC4;ZWINT;NCAPH;SMC2;SGO1;CCNA2; CENPE;SGO2; CNB1;KIF18A;CENPO;KIF20A;DNA2;SPDL1;FBXO5;BUB1
Cell Cycle	R-HSA-1640170	28	1,11E-16	ZWILCH;NCAPG;BUB1B;MCM10;SMC4;NCAPH;SMC2;CCNB1;EXO1;SPDL1;FBXO5;BUB1;PLK4;CDC7; KIF23;MASTL; WINT;SGO1;CCNA2;CENPE;SGO2;KIF18A;CENPO;KIF20A;DNA2
M Phase	R-HSA-68886	20	2,22E-16	PLK4;ZWILCH;NCAPG;BUB1B;KIF23;MASTL;SMC4;ZWINT;NCAPH;SMC2;SGO1;SGO2;CENPE;KIF18A; CNB1;CENPO;KIF20A;SPDL1;FBXO5;BUB1
Mitotic Prometaphase	R-HSA-68877	16	4,44E-16	PLK4;ZWILCH;NCAPG;BUB1B;SMC4;ZWINT;NCAPH;SMC2;SGO1;SGO2;CENPE;KIF18A;CCNB1;CENPO; SPDL1;BUB1
Cell Cycle Checkpoints	R-HSA-69620	16	3,19E-14	ZWILCH;BUB1B;MCM10;CDC7;ZWINT;SGO1;CCNA2;SGO2;CENPE;KIF18A;CCNB1;EXO1;CENPO;DNA2; SPDL1;BUB1
Amplification of signal from unattached kinetochores via a MAD2 inhibitory signal	R-HSA-141444	10	8,51E-12	SGO1;CENPE;SGO2;KIF18A;ZWILCH;BUB1B;CENPO;SPDL1;BUB1;ZWINT
Amplification of signal from the kinetochores	R-HSA-141424	10	8,51E-12	SGO1;CENPE;SGO2;KIF18A;ZWILCH;BUB1B;CENPO;SPDL1;BUB1;ZWINT
Resolution of Sister Chromatid Cohesion	R-HSA-2500257	11	1,11E-11	SGO1;CENPE;SGO2;KIF18A;CCNB1;ZWILCH;BUB1B;CENPO;SPDL1;BUB1;ZWINT
Mitotic Spindle Checkpoint	R-HSA-69618	10	4,25E-11	SGO1;CENPE;SGO2;KIF18A;ZWILCH;BUB1B;CENPO;SPDL1;BUB1;ZWINT
EML4 and NUDC in mitotic spindle formation	R-HSA-9648025	10	9,75E-11	SGO1;CENPE;SGO2;KIF18A;ZWILCH;BUB1B;CENPO;SPDL1;BUB1;ZWINT
Mitotic Metaphase and Anaphase	R-HSA-2555396	12	5,31E-10	SGO1;CENPE;SGO2;KIF18A;CCNB1;ZWILCH;BUB1B;FBXO5;CENPO;SPDL1;BUB1;ZWINT
RHO GTPases Activate Formins	R-HSA-5663220	10	7,14E-10	SGO1;CENPE;SGO2;KIF18A;ZWILCH;BUB1B;CENPO;SPDL1;BUB1;ZWINT
Condensation of Prometaphase Chromosomes	R-HSA-2514853	5	6,62E-09	CCNB1;NCAPG;SMC4;NCAPH;SMC2
Mitotic Anaphase	R-HSA-68882	11	7,05E-09	SGO1;CENPE;SGO2;KIF18A;CCNB1;ZWILCH;BUB1B;CENPO;SPDL1;BUB1;ZWINT
Separation of Sister Chromatids	R-HSA-2467813	10	9,08E-09	SGO1;CENPE;SGO2;KIF18A;ZWILCH;BUB1B;CENPO;SPDL1;BUB1;ZWINT
Signaling by Rho GTPases	R-HSA-194315	15	1,73E-07	ARHGAP11A;ARHGAP11B;ZWILCH;BUB1B;ZWINT;SGO1;SGO2;CENPE;ANLN;DEPDC1B;KIF18A; TRA2B;CENPO;SPDL1;BUB1
Signaling by Rho GTPases, Miro GTPases and RHOBTB3	R-HSA-9716542	15	2,30E-07	ARHGAP11A;ARHGAP11B;ZWILCH;BUB1B;ZWINT;SGO1;SGO2;CENPE;ANLN;DEPDC1B;KIF18A; TRA2B;CENPO;SPDL1;BUB1
Kinesins	R-HSA-983189	6	4,66E-07	CENPE;KIF18A;KIF23;KIF20A;KIF11
RHO GTPase Effectors	R-HSA-195258	10	1,02E-06	SGO1;CENPE;SGO2;KIF18A;ZWILCH;BUB1B;CENPO;SPDL1;BUB1;ZWINT
COPI-dependent Golgi-to-ER retrograde traffic	R-HSA-6811434	6	6,23E-06	CENPE;KIF18A;KIF23;KIF20A;KIF11
Mitotic G1 phase and G1/S transition	R-HSA-453279	7	8,38E-06	CCNA2;CCNB1;MCM10;CDC7;FBXO5
Golgi-to-ER retrograde transport	R-HSA-8856688	6	3,82E-05	CENPE;KIF18A;KIF23;KIF20A;KIF11
G1/S Transition	R-HSA-69206	6	4,11E-05	CCNA2;CCNB1;MCM10;CDC7;FBXO5
TP53 Regulates Transcription of Genes Involved in G1 Cell Cycle Arrest	R-HSA-6804116	3	8,82E-05	CCNA2;E2F7
Factors involved in megakaryocyte development and platelet production	R-HSA-983231	6	1,67E-04	CENPE;KIF18A;KIF23;KIF20A;KIF11
TP53 Regulates Transcription of Cell Cycle Genes	R-HSA-6791312	4	1,70E-04	CCNA2;CCNB1;E2F7
Transcriptional Regulation by TP53	R-HSA-3700989	9	1,90E-04	FANCI;CCNA2;CCNB1;EXO1;DNA2;E2F7
Intra-Golgi and retrograde Golgi-to-ER traffic	R-HSA-6811442	6	3,11E-04	CENPE;KIF18A;KIF23;KIF20A;KIF11
MHC class II antigen presentation	R-HSA-2132295	5	4,05E-04	CENPE;KIF18A;KIF23;KIF20A;KIF11
Regulation of APC/C activators between G1/S and early anaphase	R-HSA-176408	4	4,27E-04	CCNA2;CCNB1;BUB1B;FBXO5;BUB1
G2/M Checkpoints	R-HSA-69481	5	4,84E-04	CCNB1;EXO1;MCM10;CDC7;DNA2
Diseases of DNA repair	R-HSA-9675135	3	5,31E-04	NEIL3;EXO1;DNA2
Phosphorylation of Emi1	R-HSA-176417	2	5,42E-04	CCNB1;FBXO5
APC/C-mediated degradation of cell cycle proteins	R-HSA-174143	4	6,27E-04	CCNA2;CCNB1;BUB1B;FBXO5;BUB1
Regulation of mitotic cell cycle	R-HSA-453276	4	6,27E-04	CCNA2;CCNB1;BUB1B;FBXO5;BUB1
MASTL Facilitates Mitotic Progression	R-HSA-2465910	2	1,21E-03	CCNB1;MASTL
Condensation of Prophase Chromosomes	R-HSA-2299718	3	1,65E-03	CCNB1;SMC4;SMC2
Mitotic Telophase/Cytokinesis	R-HSA-68884	2	1,87E-03	KIF23;KIF20A

Mitotic Prophase	R-HSA-68875	4	2,54E-03	CCNB1;MASTL;SMC4;SMC2
DNA Replication	R-HSA-69306	4	3,05E-03	CCNA2;MCM10;CDC7;DNA2
Transcription of E2F targets under negative control by p107 (RBL1) and p130 (RBL2) in complex with HDAC1	R-HSA-1362300	2	3,28E-03	CCNA2
RHOA GTPase cycle	R-HSA-8980692	4	4,06E-03	ARHGAP11A;ARHGAP11B;ANLN;DEPDC1B
APC/C:Cdc20 mediated degradation of mitotic proteins	R-HSA-176409	3	4,10E-03	CCNA2;CCNB1;BUB1B;BUB1
NEIL3-mediated resolution of ICLs	R-HSA-9636003	1	4,19E-03	NEIL3
Defective Base Excision Repair Associated with NEIL3	R-HSA-9629232	1	4,19E-03	NEIL3
Activation of APC/C and APC/C:Cdc20 mediated degradation of mitotic proteins	R-HSA-176814	3	4,25E-03	CCNA2;CCNB1;BUB1B;BUB1
Polo-like kinase mediated events	R-HSA-156711	2	4,30E-03	CCNB1
Defective HDR through Homologous Recombination (HRR) due to PALB2 loss of function	R-HSA-9701193	2	4,67E-03	EXO1;DNA2
Defective HDR through Homologous Recombination Repair (HRR) due to PALB2 loss of BRCA2/RAD51/RAD51C binding function	R-HSA-9704646	2	4,67E-03	EXO1;DNA2
Defective HDR through Homologous Recombination Repair (HRR) due to PALB2 loss of BRCA1 binding function	R-HSA-9704331	2	4,67E-03	EXO1;DNA2
Diseases of DNA Double-Strand Break Repair	R-HSA-9675136	2	4,67E-03	EXO1;DNA2
G2/M DNA damage checkpoint	R-HSA-69473	3	4,89E-03	CCNB1;EXO1;DNA2
TP53 Regulates Transcription of DNA Repair Genes	R-HSA-6796648	3	6,33E-03	FANCI
Processing of DNA double-strand break ends	R-HSA-5693607	3	6,53E-03	CCNA2;EXO1;DNA2
Resolution of D-loop Structures through Synthesis-Dependent Strand Annealing (SDSA)	R-HSA-5693554	2	7,18E-03	EXO1;DNA2
Regulation of TP53 Activity through Phosphorylation	R-HSA-6804756	3	7,57E-03	CCNA2;EXO1;DNA2
Cyclin A/B1/B2 associated events during G2/M transition	R-HSA-69273	2	8,13E-03	CCNA2;CCNB1
Activation of the pre-replicative complex	R-HSA-68962	2	1,02E-02	MCM10;CDC7
Resolution of D-loop Structures through Holliday Junction Intermediates	R-HSA-5693568	2	1,02E-02	EXO1;DNA2
NOTCH3 Intracellular Domain Regulates Transcription	R-HSA-9013508	2	1,02E-02	DLGAP5
Resolution of D-Loop Structures	R-HSA-5693537	2	1,07E-02	EXO1;DNA2
G0 and Early G1	R-HSA-1538133	2	1,13E-02	CCNA2
HDR through Single Strand Annealing (SSA)	R-HSA-5685938	2	1,19E-02	EXO1;DNA2
Activation of ATR in response to replication stress	R-HSA-176187	2	1,19E-02	MCM10;CDC7
G2/M Transition	R-HSA-69275	4	1,22E-02	PLK4;CCNA2;CCNB1
Mitotic G2-G2/M phases	R-HSA-453274	4	1,26E-02	PLK4;CCNA2;CCNB1
Presynaptic phase of homologous DNA pairing and strand exchange	R-HSA-5693616	2	1,30E-02	EXO1;DNA2
G1/S-Specific Transcription	R-HSA-69205	2	1,43E-02	FBXO5
Homologous DNA Pairing and Strand Exchange	R-HSA-5693579	2	1,49E-02	EXO1;DNA2
HDR through Homologous Recombination (HRR) or Single Strand Annealing (SSA)	R-HSA-5693567	3	1,55E-02	CCNA2;EXO1;DNA2
Transcriptional Regulation by E2F6	R-HSA-8953750	2	1,62E-02	CDC7
Mitotic Metaphase/Anaphase Transition	R-HSA-68881	1	1,66E-02	FBXO5
Homology Directed Repair	R-HSA-5693538	3	1,75E-02	CCNA2;EXO1;DNA2
DNA Repair	R-HSA-73894	5	1,93E-02	FANCI;CCNA2;NEIL3;EXO1;DNA2
Chromosome Maintenance	R-HSA-73886	3	2,04E-02	CCNA2;CENPO;DNA2
Phosphorylation of proteins involved in the G2/M transition by Cyclin A:Cdc2 complexes	R-HSA-170145	1	2,08E-02	CCNA2
G2 Phase	R-HSA-68911	1	2,89E-02	CCNA2
G2/M DNA replication checkpoint	R-HSA-69478	1	2,89E-02	CCNB1
Signaling by NOTCH3	R-HSA-9012852	2	2,90E-02	DLGAP5
Diseases of Base Excision Repair	R-HSA-9605308	1	3,30E-02	NEIL3
DNA Double-Strand Break Repair	R-HSA-5693532	3	3,38E-02	CCNA2;EXO1;DNA2
Extension of Telomeres	R-HSA-180786	2	3,43E-02	CCNA2;DNA2

HDR through Homologous Recombination (HRR)	R-HSA-5685942	2	3,70E-02	EXO1;DNA2
E2F-enabled inhibition of pre-replication complex formation	R-HSA-113507	1	3,71E-02	CCNB1
Activation of NIMA Kinases NEK9, NEK6, NEK7	R-HSA-2980767	1	3,71E-02	CCNB1
Cdc20:Phospho-APC/C mediated degradation of Cyclin A	R-HSA-174184	2	3,79E-02	CCNA2;BUB1B;BUB1
APC:Cdc20 mediated degradation of cell cycle proteins prior to satisfation of the cell cycle checkpoint	R-HSA-179419	2	3,89E-02	CCNA2;BUB1B;BUB1
Regulation of TP53 Activity	R-HSA-5633007	3	3,90E-02	CCNA2;EXO1;DNA2
RHOB GTPase cycle	R-HSA-9013026	2	3,98E-02	ANLN;DEPDC1B
RHOC GTPase cycle	R-HSA-9013106	2	4,37E-02	ANLN;DEPDC1B
RHO GTPase cycle	R-HSA-9012999	5	4,37E-02	ARHGAP11A;ARHGAP11B;ANLN;DEPDC1B;TRA2B

Table S7. Gene ontology (GO) enrichment analysis for the top 50 genes positively correlated with *SPDL*1 in colorectal cancer.

GO TERMS	GO ID	COUNT	P VALUE	GENES
**Biological process**				
Cell division	GO:0051301	20	4,57E-20	HELLS, ZWILCH, NCAPG, BUB1B, CDC7, KIF11, MASTL, SMC4, KNSTRN, NCAPH, ZWINT, SMC2, SGO1, CCNA2, CENPE, SGO2, CCNB1, SPDL1, FBXO5, BUB1
Sister chromatid cohesion	GO:0007062	10	1,18E-11	SGO1, CENPE, SGO2, KIF18A, ZWILCH, BUB1B, CENPO, SPDL1, BUB1, ZWINT
Mitotic nuclear division	GO:0007067	11	1,65E-09	SGO1, CCNA2, ANLN, HELLS, ZWILCH, BUB1B, KIF11, FBXO5, MASTL, BUB1, CEP55
Mitotic chromosome condensation	GO:0007076	5	7,84E-08	NUSAP1, NCAPG, SMC4, NCAPH, SMC2
Mitotic cytokinesis	GO:0000281	5	1,32E-06	ANLN, NUSAP1, KIF23, KIF20A, CEP55
Mitotic cell cycle checkpoint	GO:0007093	5	1,99E-06	ZWILCH, BUB1B, TTK, BUB1, ZWINT
Mitotic metaphase plate congression	GO:0007080	5	3,62E-06	CENPE, KIF18A, CCNB1, SPDL1, CEP55
Meiotic chromosome segregation	GO:0045132	3	4,78E-05	SGO1, SMC4, SMC2
Mitotic sister chromatid segregation	GO:0000070	4	4,82E-05	NUSAP1, KNSTRN, SMC4, ZWINT
Microtubule-based movement	GO:0007018	5	8,32E-05	CENPE, KIF18A, KIF23, KIF20A, KIF11
Protein localization to kinetochore	GO:0034501	3	3,55E-04	BUB1B, TTK, SPDL1
Mitotic G2 DNA damage checkpoint	GO:0007095	3	8,20E-04	CCNA2, FANCI, SLF1
Spindle organization	GO:0007051	3	9,36E-04	TTK, KIF11, KNSTRN
Chromosome segregation	GO:0007059	4	9,64E-04	SGO1, CENPE, KIF11, KNSTRN
DNA replication	GO:0006260	5	9,91E-04	RRM1, EXO1, MCM10, CDC7, DNA2
Mitotic spindle assembly checkpoint	GO:0007094	3	1,47E-03	BUB1B, TTK, BUB1
Retrograde vesicle-mediated transport, Golgi to ER	GO:0006890	4	1,66E-03	CENPE, KIF18A, KIF23, KIF11
Antigen processing and presentation of exogenous peptide antigen via MHC class II	GO:0019886	4	2,30E-03	CENPE, KIF18A, KIF23, KIF11
Mitotic spindle organization	GO:0007052	3	3,31E-03	CCNB1, TTK, KIF11
DNA replication initiation	GO:0006270	3	3,76E-03	SLF1, MCM10, CDC7
G2/M transition of mitotic cell cycle	GO:0000086	4	7,03E-03	PLK4, CCNB1, MELK, MASTL
Kinetochore organization	GO:0051383	2	8,55E-03	SMC4, SMC2
Mitotic chromosome movement towards spindle pole	GO:0007079	2	1,14E-02	CENPE, DLGAP5
Meiotic chromosome condensation	GO:0010032	2	1,42E-02	SMC4, SMC2
Negative regulation of ubiquitin-protein ligase activity involved in mitotic cell cycle	GO:0051436	3	1,75E-02	CCNB1, BUB1B, FBXO5
Positive regulation of ubiquitin-protein ligase activity	GO:0051437	3	1,99E-02	CCNB1, BUB1B, FBXO5

involved in regulation of mitotic cell cycle transition				
Cell proliferation	GO:0008283	5	2,03E-02	MELK, BUB1B, MCM10, BUB1, DLGAP5
Trophoblast giant cell differentiation	GO:0060707	2	3,38E-02	PLK4, E2F7
G1/S transition of mitotic cell cycle	GO:0000082	3	3,44E-02	MCM10, CDC7, FBXO5
Centriole replication	GO:0007099	2	4,48E-02	PLK4, SASS6
Peptidyl-serine phosphorylation	GO:0018105	3	4,97E-02	TTK, CDC7, MASTL
**Cellular components**				
Kinetochore	GO:0000776	9	4,93E-11	SGO1, CENPE, KIF18A, ZWILCH, BUB1B, TTK, KNSTRN, BUB1, ZWINT
Condensed chromosome kinetochore	GO:0000777	9	8,85E-11	SGO1, CENPE, SGO2, ZWILCH, BUB1B, CENPO, KNSTRN, BUB1, ZWINT
Nucleoplasm	GO:0005654	26	9,06E-09	MCM10, HNRNPR, SMC4, SMC2, CCNB1, EXO1, TRA2B, FBXO5, BUB1, E2F7, FANCI, WDHD1, RRM1, KIF23, CDC7, MASTL, SGO1, CCNA2, SGO2, MMS22L, ANLN, NEIL3, DEPDC1, KIF20A, CENPO, DNA2
Condensin complex	GO:0000796	4	1,94E-07	NCAPG, SMC4, NCAPH, SMC2
Spindle	GO:0005819	7	9,47E-07	NUSAP1, KIF23, TTK, KIF20A, KIF11, FBXO5, SHCBP1
Centrosome	GO:0005813	10	2,05E-06	SGO1, PLK4, SLF1, CCNB1, CKAP2L, SASS6, NCAPG, KIF23, MASTL, CEP55
Spindle pole	GO:0000922	6	1,19E-05	SGO1, CCNB1, CKAP2L, KIF11, SPDL1, KNSTRN
Kinesin complex	GO:0005871	5	1,33E-05	CENPE, KIF18A, KIF23, KIF20A, KIF11
Cytosol	GO:0005829	23	2,20E-05	ARHGAP11A, PLK4, ARHGAP11B, RRM1, ZWILCH, NCAPG, BUB1B, KIF23, KIF11, SMC4, NCAPH, ZWINT, SMC2, SGO1, CENPE, SGO2, KIF18A, DEPDC1B, CCNB1, CENPO, SPDL1, FBXO5, BUB1
Cytoplasm	GO:0005737	28	1,46E-04	NCAPG, BUB1B, MCM10, TTK, KIF11, SMC4, SMC2, KIAA1524, CCNB1, EXO1, NUSAP1, FBXO5, BUB1, DLGAP5, FANCI, WDHD1, SLF1, RRM1, CKAP2L, CDC7, MASTL, KNSTRN, SHCBP1, ZWINT, SGO1, CCNA2, CENPE, KIF18A
Condensed chromosome outer kinetochore	GO:0000940	3	2,04E-04	CENPE, BUB1B, SPDL1
Nucleus	GO:0005634	28	2,82E-04	NCAPG, MCM10, SMC4, NCAPH, SMC2, CCNB1, EXO1, TRA2B, NUSAP1, FBXO5, SPDL1, E2F7, DLGAP5, HELLS, SLF1, KIF23, CDC7, MASTL, KNSTRN, ZWINT, SGO1, CCNA2, CENPE, KIF18A, NEIL3, MELK, DEPDC1, DNA2
Midbody	GO:0030496	5	4,29E-04	CENPE, KIF23, KIF20A, SHCBP1, CEP55
Chromosome, centromeric region	GO:0000775	4	5,12E-04	SGO1, CENPE, SGO2, HELLS
Microtubule	GO:0005874	6	1,58E-03	CENPE, KIF18A, NUSAP1, KIF23, KIF20A, KIF11
Microtubule cytoskeleton	GO:0015630	4	6,29E-03	CENPE, KIF18A, CDC7, KNSTRN
Intercellular bridge	GO:0045171	3	6,48E-03	KIF23, CDC7, CEP55
Cleavage furrow	GO:0032154	3	7,37E-03	PLK4, MASTL, CEP55
Microtubule organizing center	GO:0005815	4	8,52E-03	KIF18A, BUB1B, SPDL1, DLGAP5
Condensed nuclear chromosome outer kinetochore	GO:0000942	2	1,09E-02	CCNB1, BUB1
Deuterosome	GO:0098536	2	1,36E-02	PLK4, SASS6
Condensed chromosome, centromeric region	GO:0000779	2	1,91E-02	SGO1, CENPE
Mitotic spindle midzone	GO:1990023	2	2,44E-02	CENPE, KIF18A
Chromosome	GO:0005694	3	3,37E-02	CENPE, NUSAP1, SMC4
Centriole	GO:0005814	3	3,85E-02	PLK4, SASS6, CEP55
**Molecular function**				
Protein binding	GO:0005515	43	7,15E-07	
ATP binding	GO:0005524	17	2,63E-06	PLK4, HELLS, RRM1, BUB1B, TTK, KIF23, CDC7, KIF11, MASTL, SMC4, SMC2, CENPE, KIF18A, MELK, KIF20A, DNA2, BUB1
Microtubule motor activity	GO:0003777	5	8,43E-05	CENPE, KIF18A, KIF23, KIF20A, KIF11
Microtubule binding	GO:0008017	6	3,32E-04	CENPE, KIF18A, NUSAP1, KIF23, KIF20A, KIF11
Protein serine/threonine kinase activity	GO:0004674	7	7,31E-04	PLK4, MELK, BUB1B, TTK, CDC7, MASTL, BUB1
Kinetochore binding	GO:0043515	2	1,16E-02	CENPE, SPDL1
ATPase activity	GO:0016887	4	1,60E-02	CENPE, KIF23, KIF20A, DNA2

Protein kinase binding	GO:0019901	5	2,34E-02	CCNA2, CCNB1, KIF20A, KIF11, FBXO5
GTPase activator activity	GO:0005096	4	4,71E-02	ARHGAP11A, ARHGAP11B, DEPDC1B, DEPDC1
ATP-dependent microtubule motor activity, plus-end-directed	GO:0008574	2	4,82E-02	KIF18A, KIF11

Table S8. Missing data on testing variables.

Variable	Missing data	
	**TMA cohort (%)**	**TCGA cohort (%)**
survival time	8 (10.7)	4 (1.5)
survival status	0 (0)	4 (1.5)
grade	2 (2.7)	-
pN	3 (4.0)	0 (0)
pM	6 (8.0)	6 (2.2)
VI	40 (53.3)	-
PNI	50 (66.7)	-

Abbreviations: pM - distant metastasis, pN - regional lymph node, PNI - perineural invasion, TCGA - The Cancer Genome Atlas, TMA - tissue microarray, VI - vascular invasion. "-" indicates lack of data on variable.

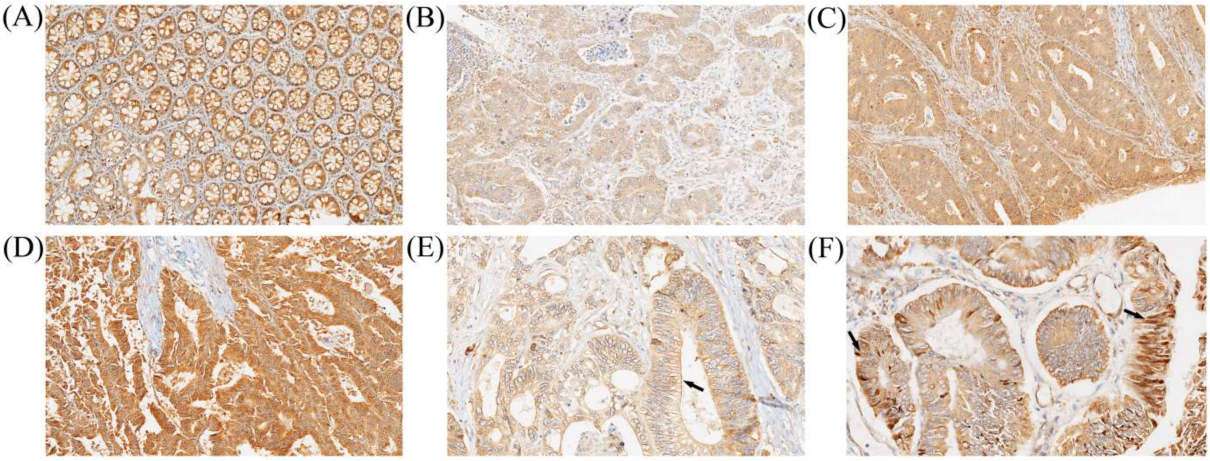

Figure S1. Representative immunohistochemical SPDL1 protein stains showing (A) moderately positive staining in normal tissue; original magnifications: 10×; (B) weakly positive staining in CRC; original magnifications: 10×; (C) moderately positive staining in CRC; original magnifications: 10×; (D) strongly positive staining in CRC; original magnifications: 10×; (E) membrane staining in addition to cytoplasmic staining in CRC (arrow), original magnifications: 20×; (F) several positive nuclei in addition to cytoplasmic staining in CRC (arrows); original magnifications: 20×.

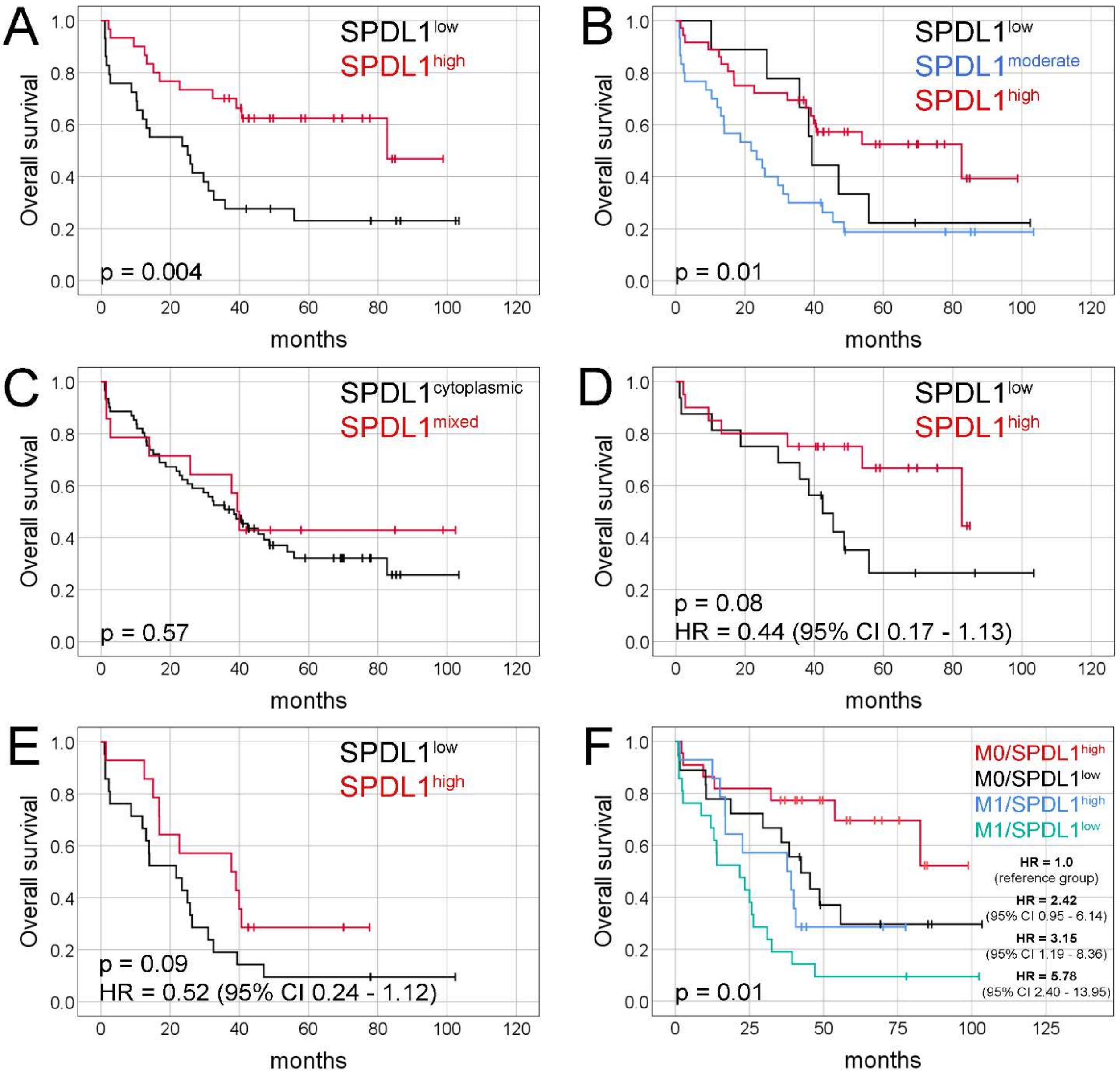

Figure S2. Kaplan-Meier survival curves and log-rank test for overall survival of CRC patients in TMA cohort according to SPDL1 protein expression (A) when the analysis was restricted to individuals with complete data (n=59); (B) after splitting the IRS scores into three groups: low (n = 9; IRS < 5), moderate (n = 30; IRS ≥ 5 < 7.5) and high (n =36; IRS ≥ 7.5); (C) when the analysis was based on the subcellular staining pattern: purely cytoplasmic (n = 61) or mixed pattern (n= 14; nuclear and cytoplasmic or membranous and cytoplasmic); (D) when the analysis was restricted to AJCC TNM stage II-III CRCs (n = 36; the cut-off 7.5); (E) when the analysis was restricted to AJCC TNM stage IV CRCs (n = 35; the cut-off 7.5); (F) when stratified by metastatic (pM) status [M0/SPDL1^{high}: patient without distant metastasis and SPDL1-high expression, n = 22; M0/SPDL1^{low}: patient without distant metastasis and SPDL1-low expression, n = 18; M1/SPDL1^{high}: patient with distant metastasis and SPDL1-high expression, n = 14; M1/SPDL1^{low}: patient with distant metastasis and SPDL1-low expression, n = 21; the p value shown has been pooled over strata]. HR: hazard ratio (Cox proportional hazards model), CI: confidence interval.

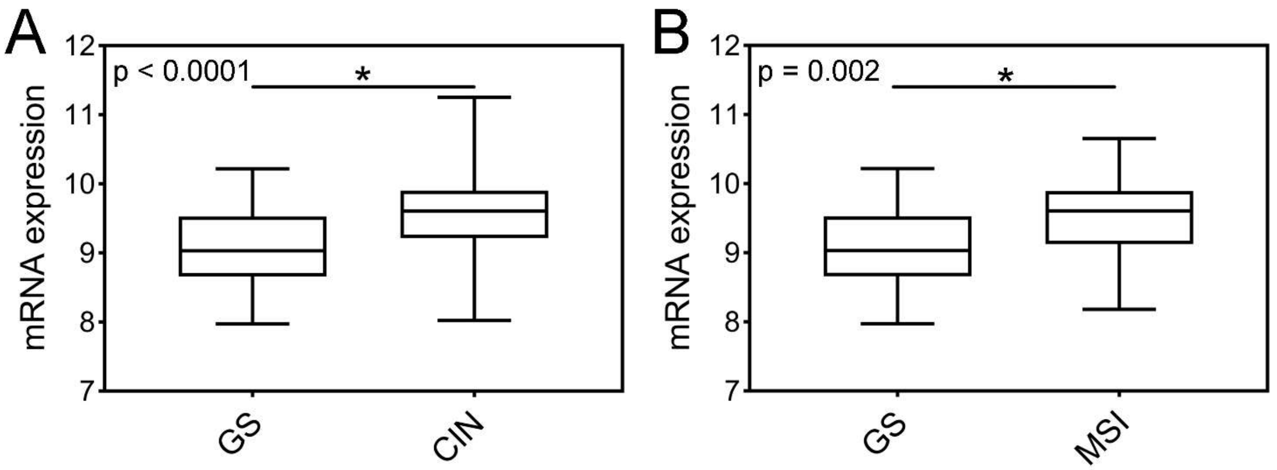

Figure S3. The expression level of *SPDL1* in (A) chromosomal instability (CIN subtype) colorectal cancer; (B) microsatellite instability (MSI subtype) colorectal cancer compared to genomically stable (GS subtype) tumors. The error bars present the range from minimum to maximum values of data. Asterisks (*) indicate statistically significant differences (p < 0.05).

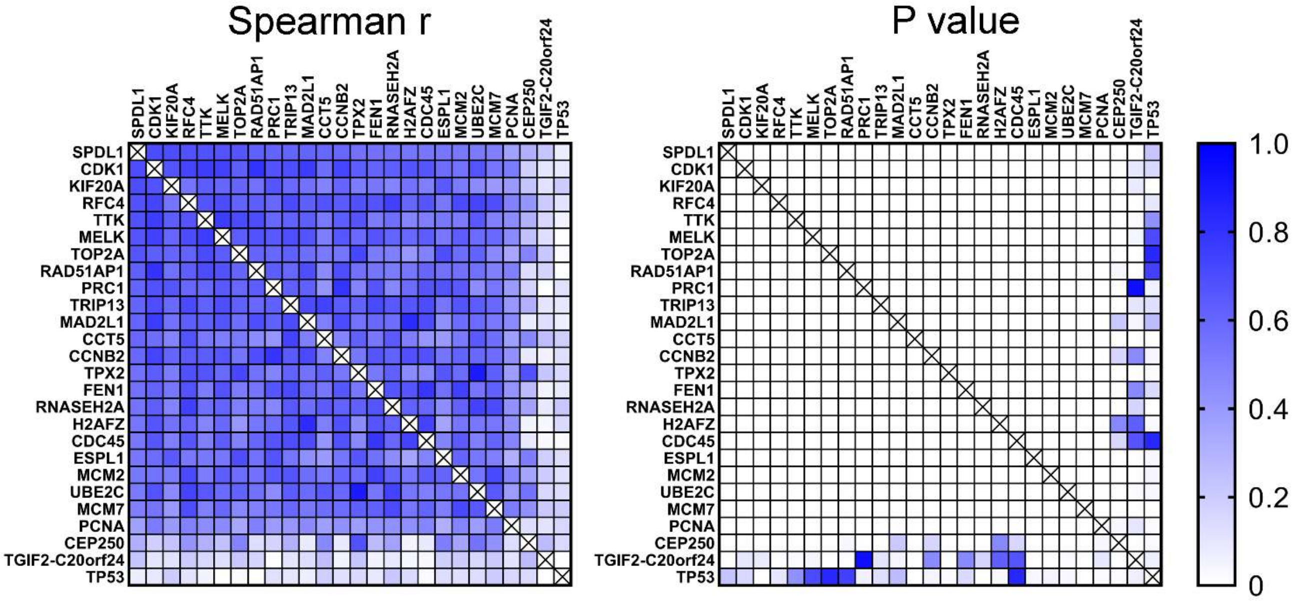

Figure S4. Heatmaps presenting Spearman correlation and P value between the expression of *SPDL1* and 25 genes associated with functional aneuploidy (CIN25 signature).

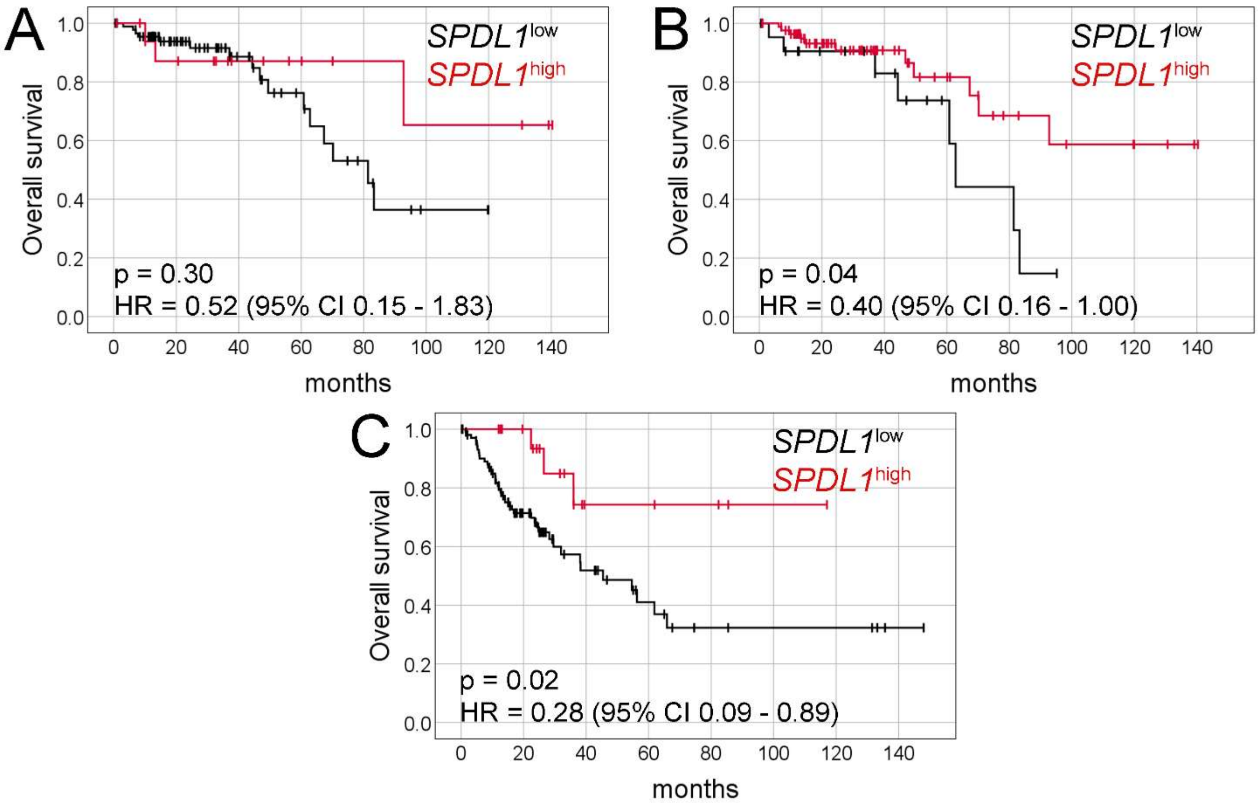

Figure S5. Kaplan-Meier survival curves and log-rank test for overall survival in the TCGA cohort based on *SPDL1* mRNA expression in distinct AJCC TNM stage CRC patients (A) stage II with the cut-off 9.992 (n = 107); (B) stage II with the cut-off 9.02 (n = 107); (C) stage III-IV with the cut-off 9.992 (n = 123). HR: hazard ratio (Cox proportional hazards model). CI: confidence interval.
